# Supplementary material for: Restructured membrane contacts rewire organelles for human cytomegalovirus infection
Source: Nat Commun. 2022 Aug 11;13:4720. doi: 10.1038/s41467-022-32488-6 (PMC9366835; doi:10.1038/s41467-022-32488-6)
Supplement: Supplementary file 1 — Supplementary Information [file 41467_2022_32488_MOESM1_ESM.pdf]

SUPPLEMENTARY TABLES

Supplementary Table 1. *Library of membrane contact site proteins included in MCS-PRM, Related to Figure 1.*

MCS proteins were chosen to be included in our assay based on having reported functions linked to a specific MCS localization. A report of a localization of a protein at an inter-organelle junction was not deemed sufficient for including that protein in our MCS-PRM assay, without additional knowledge of the specific contribution of that protein to the formation of the organelle contact or a downstream function of the MCS. Several proteins that are only transiently enriched at MCSs (*e.g.*, DRP1, DNM2, INF2 and NPC1) are included in our assay, given the defined functions of these proteins dependent on their MCS enrichment (*e.g.*, ER-mediated mitochondrial fission and ER-endosome cholesterol transfer, respectively). For the MCS proteins included in our assay, listed below are their defined tethering partners, known MCS localizations, contact-dependent functions, and citations for their characterized biological mechanisms. References can be found at the end of this document.

Protein	*Uniprot #*	Organelle Localization	MCS	Process	MCS partners	Citations
**MFN1**	*Q8IWA4*	Mito	Mito-ER	Mitochondrial fusion; Mitochondrial fission	VAPA; VAPB; MFN2	1,2
**MFN2**	*O95140*	Mito; ER	Mito-ER	Mitochondrial fusion; Mitochondrial fission	VAPA; VAPB; MFN1	1,2
**VDAC1**	*P21796*	Mito	Mito-ER	Calcium transfer; Mitochondrial integrity	HSPA9; ITPR3	3
**VDAC2**	*P45880*	Mito	Mito-ER	Calcium transfer; Mitochondrial integrity	HSPA9; ITPR3	3
**VDAC3**	*Q9Y277*	Mito	Mito-ER	Calcium transfer; Mitochondrial integrity	HSPA9; ITPR3	3
**ITPR1**	*Q14643*	Mito	Mito-ER	Calcium transfer; Mitochondrial integrity	HSPA9; VDAC1; VDAC2; VDAC3	3
**ITPR3**	*Q14573*	Mito	Mito-ER	Calcium transfer; Mitochondrial integrity	HSPA9; VDAC1; VDAC2; VDAC3	3
**RRBP1**	*Q9P2E9*	ER	Mito-ER	Protein translation; Organelle tethering	SYNJ2BP	4
**SYNJ2BP (OMP25)**	*P57105*	Mito	Mito-ER	Protein translation; Organelle tethering	RRBP1	4
**FIS1**	*Q9Y3D6*	Mito; Perox	Mito-ER	Mitochondrial fission; Apoptosis; Vesicle maturation	DNM1L; MFF; DNM2; BAP31	5,6
**MFF**	*Q9GZY8*	Mito; Perox	Mito-ER	Mitochondrial fission	DNM1L; FIS1; DNM2; MFN1; MFN2	2,5
**TBC1D15**	*Q8TC07*	Cytoplasm; Endosome	Mito-Lysosome; Mito-ER	Mitochondrial fission; Vesicle maturation	RAB7A; FIS1; STARD3; ANXA6	6,7
**OPA1**	*O60313*	Mito	Mito OMM-IMM; Mito-ER	Mitochondrial integrity; Mitochondrial fusion	MFN1; MFN2; MICOS & SAMM50 complex	8–10
**DNM1L (DRP1)**	*O00429*	Cytoplasm; Mito	Mito-ER	Mitochondrial fission	MFF; FIS1; DNM2	5
**DNM2**	*P50570*	Cytoplasm; Mito	Mito-ER	Mitochondrial fission	MFF; FIS1; DNM1L	11
**INF2**	*Q27J81*	ER	Mito-ER	Mitochondrial fission; Calcium transfer	MYOIIA; DRP1	12
**MIRO1**	*Q8IXI2*	Cytoplasm; Mito; Perox	Mito-ER; Mito OMM-IMM	Mitochondrial integrity; Mitochondrial fission; Mitochondrial transport	MICOS; KIF5B; MIRO2	13,14
**MIRO2**	*Q8IXI1*	Cytoplasm; Mito; Perox	Mito-ER; Mito OMM-IMM	Mitochondrial integrity; Mitochondrial fission; Mitochondrial transport	MICOS; MIRO1	14
**HSPA9 (GRP75)**	*P38646*	Cytoplasm; Mito	Mito-ER	Calcium transfer; Mitochondrial integrity	ITPR1; ITPR3; VDAC1; VDAC2; VDAC3	3,15
**PTPIP51 (RMDN3)**	*Q96TC7*	Cytoplasm; Mito	Mito-ER	Calcium transfer; Autophagosome biogenesis	VAPB; TADBP; GSK3B	16–18
**TADBP (TDP-43)**	*Q13148*	Nucleus	Mito-ER	Organelle tethering; calcium transfer	PTPIP51; VAPB; GSK3B	17
**GSK3B**	*P49841*	Cytoplasm	Mito-ER	Organelle tethering; calcium transfer	PTPIP51; VAPB; TDP-43	17
**BAP31 (BCAP31)**	*P51572*	ER	Mito-ER	Apoptosis; Mitochondrial integrity; Calcium transfer	FIS1; TOM40; PACS2	19–22

**PACS2**	*Q86VP3*	Cytoplasm; Mito	Mito-ER	Apoptosis; Autophagosome biogenesis	BAP31; STX17	20,23,24
**VAMP8**	*Q9BV40*	Endosome; Autophagosome	Mito-ER; Lysosome-ER; Mito-Lysosome	Autophagosome biogenesis	STX17; ATG14	23,25
**VMP1**	*Q96GC9*	ER	Mito-ER, ER-LD, ER-Endosome	Autophagosome biogenesis	SERCA	26–28
**ATG14 (BAKOR)**	*Q6ZNE5*	Mitochondria; Autophagosome	Mito-ER; ER-Lysosome; Mito-Lysosome	Autophagosome biogenesis	VAMP8; STX17	23,25
**STX17**	*P56962*	Mitochondria; Autophagosome	Mito-ER	Autophagosome biogenesis	ATG14; VAMP8	23
**VAPA**	*Q9P0L0*	ER	Endosome-ER; Golgi-ER; Peroxisome-ER; Mito-ER; PM-ER	Mitochondrial fission; Calcium transfer; Lipid transfer; Mitochondrial fusion; Autophagosome biogenesis; Peroxisome maintenance; Vesicle trafficking; Cholesterol transfer; Vesicle maturation	Numerous proteins with FFAT motifs	29–33
**VAPB**	*O95292*	ER	Endosome-ER; Golgi-ER; Peroxisome-ER; Mito-ER; PM-ER	Mitochondrial fission; Calcium transfer; Lipid transfer; Mitochondrial fusion; Autophagosome biogenesis; Peroxisome maintenance; Vesicle trafficking; Cholesterol transfer; Vesicle maturation	Numerous proteins with FFAT motifs	29–33
**MOSPD2**	*Q8NHP6*	ER	Mitochondria-ER; Endosome-ER; Golgi-ER	Organelle tethering	Numerous proteins with FFAT motifs	34
**STARD3**	*Q14849*	Endosome	Endosome-ER	Vesicle trafficking; Cholesterol transfer; Vesicle maturation	STARD3NL; VAPA; VAPB; ORP1L; NPC1; RAB7A; TBC1D15; ANXA1; ANXA6	7,35–38
**STARD3NL**	*O95772*	Endosome	Endosome-ER	Vesicle trafficking; Cholesterol transfer; Vesicle maturation	STARD3; VAPA; VAPB; ORP1L; NPC1; RAB7A; ANXA1	35,36,38
**NPC1**	*O15118*	Endosome	Endosome-ER	Cholesterol transfer; Vesicle maturation	STARD3; STARD3NL	35–37
**ANXA6**	*P08133*	Endosome	Endosome-ER	Cholesterol transfer; Vesicle maturation	STARD3;STARD3NL; NPC1; ORP1L; RAB7A; TBC1D15	7
**ORP1L (OSBPL1A)**	*Q9BXW6*	Endosome	Endosome-ER; Mitochondria-Lysosome-ER	Vesicle trafficking; Cholesterol transfer; Vesicle maturation; Autophagosome biogenesis	VAPA; VAPB; RAB7A; ORP5; NPC1; ANXA1	37–40
**ORP5 (OSBPL5)**	*Q9H0X9*	PM; Endosome	Endosome-ER; PM-ER	Lipid transfer	VAPA; VAPB; ORP1L	41,42
**Protrudin (ZFYVE27)**	*Q5T4F4*	Endosome	Endosome-ER	Vesicle trafficking	VAPA; VAPB; RAB7A; PDZD8; FYCO1	43,44
**PDZD8**	*Q8NEN9*	ER	Endosome-ER; Mito-Endosome	Vesicle trafficking; mitochondrial integrity	RAB7A; Protrudin	44,45
**RAB7A**	*P51149*	Endosome	Endosome-ER; Mitochondria-Lysosome	Vesicle trafficking; Vesicle maturation; Mitochondrial fission	TBC1D15; FIS1; STARD3; STARD3NL; Protrudin; TMCC1; CORO1C; PDZD8; ANXA6	6,7,44,46,47
**RAB9A**	*P51151*	Endosome	Endosome-ER	Vesicle maturation	RTN3	48
**RTN3**	*O95197*	ER	Endosome-ER	Vesicle maturation	RAB9A	48
**EGFR**	*P00533*	PM; Endosome	Endosome-ER	Cell signaling	PTPN1; ANXA1	38,49
**PTPN1 (PTP1B)**	*P18031*	ER	Endosome-ER	Cell signaling	EGFR; ANXA1	38,49

**ANXA1**	*P04083*	PM; Endosome	Endosome-ER	Cell signaling; Cholesterol transfer; MVB biogenesis	PTPN1; EGFR; ORP1L; VAPA; VAPB	38
**TMCC1**	*O94876*	ER	Endosome-ER	Vesicle fission; Vesicle sorting	CORO1C; RAB7A	47
**CORO1C**	*Q9ULV4*	Cytoplasm; Endosome	Endosome-ER	Vesicle fission; Vesicle sorting	TMCC1; RAB7A	47
**CERT (COL4A3BP)**	*Q9Y5P4*	Golgi	Golgi-ER	Lipid transfer	VAPA; VAPB	50
**ORAI1**	*Q96D31*	PM	PM-ER	Calcium transfer	VAPA; VAPB; STIM1	42,51,52
**ORP8 (OSBPL8)**	*Q9BZF1*	PM; Golgi	PM-ER; Golgi-ER	Lipid transfer	VAPA; VAPB	42
**ESYT1**	*Q9BSJ8*	ER	PM-ER	Lipid transfer; Calcium transfer		53,54
**ESYT2**	*A0FGR8*	ER	PM-ER	Lipid transfer; Calcium transfer		53,54
**STIM1**	*Q86TL2*	ER	PM-ER	Calcium transfer	STIM1	51
**STIMATE**	*Q13586*	PM	PM-ER	Calcium transfer	STIMATE	51,53
**NIR3 (PTPIPNM2)**	*Q9BZ72*	PM	PM-ER	Lipid transfer	VAPA; VAPB	55
**ACBD5**	*Q5T8D3*	Perox	Peroxisome-ER	Lipid transfer; Peroxisome membrane growth & maintenance	VAPA; VAPB	56,57
**ACBD4**	*Q8NC06*	Perox	Peroxisome-ER	Peroxisome maintenance	ACBD5; VAPB	58
**SPASTIN**	*Q9UBP0*	Lipid droplets	Peroxisome-Lipid droplet	Lipid transfer; organelle tethering	ESCRT-III; ABCD1	59
**IST1 (ESCRT III)**	*P53990*	Cytoplasm; Lipid droplets; Perox	Peroxisome-Lipid droplet	Lipid transfer	SPASTIN	59
**CHMP1B (ESCRT III)**	*Q7LBR1*	Cytoplasm; Lipid droplets; Perox	Peroxisome-Lipid droplet	Lipid transfer	SPASTIN	59
**IMMT (MIC60)**	*Q16891*	Mito	Mito OMM-IMM	Mitochondrial integrity	MICOS & SAMM50 complex; OPA1; MFN1; MFN2	60–62
**SAMM50**	*Q9Y512*	Mito	Mito OMM-IMM	Mitochondrial integrity	MICOS & SAMM50 complex; OPA1; MFN1; MFN2	10,63–65
**MTX1**	*Q13505*	Mito	Mito-ER	Mitochondrial integrity	MICOS & SAMM50 complex; OPA1; MFN1; MFN2	64
**MTX2**	*O75431*	Mito	Mito-ER	Mitochondrial integrity	MICOS & SAMM50 complex; OPA1; MFN1; MFN2	64
**APOO (MIC26)**	*Q9BUR5*	Mito	Mito OMM-IMM	Mitochondrial integrity	MICOS & SAMM50 complex; OPA1; MFN1; MFN2	63
**CHCHD3 (MIC19)**	*Q9NX63*	Mito	Mito OMM-IMM	Mitochondrial integrity	MICOS & SAMM50 complex; OPA1; MFN1; MFN2	61,64,66
**CHCHD6 (MIC25)**	*Q9BRQ6*	Mito	Mito OMM-IMM	Mitochondrial integrity	MICOS & SAMM50 complex; OPA1; MFN1; MFN2	64,65,67
**CD99 (MIC2)**	*P14209*	Mito	Mito OMM-IMM	Mitochondrial integrity	MICOS & SAMM50 complex; OPA1; MFN1; MFN2	60,62

**See all references below.*

SUPPLEMENTARY FIGURES

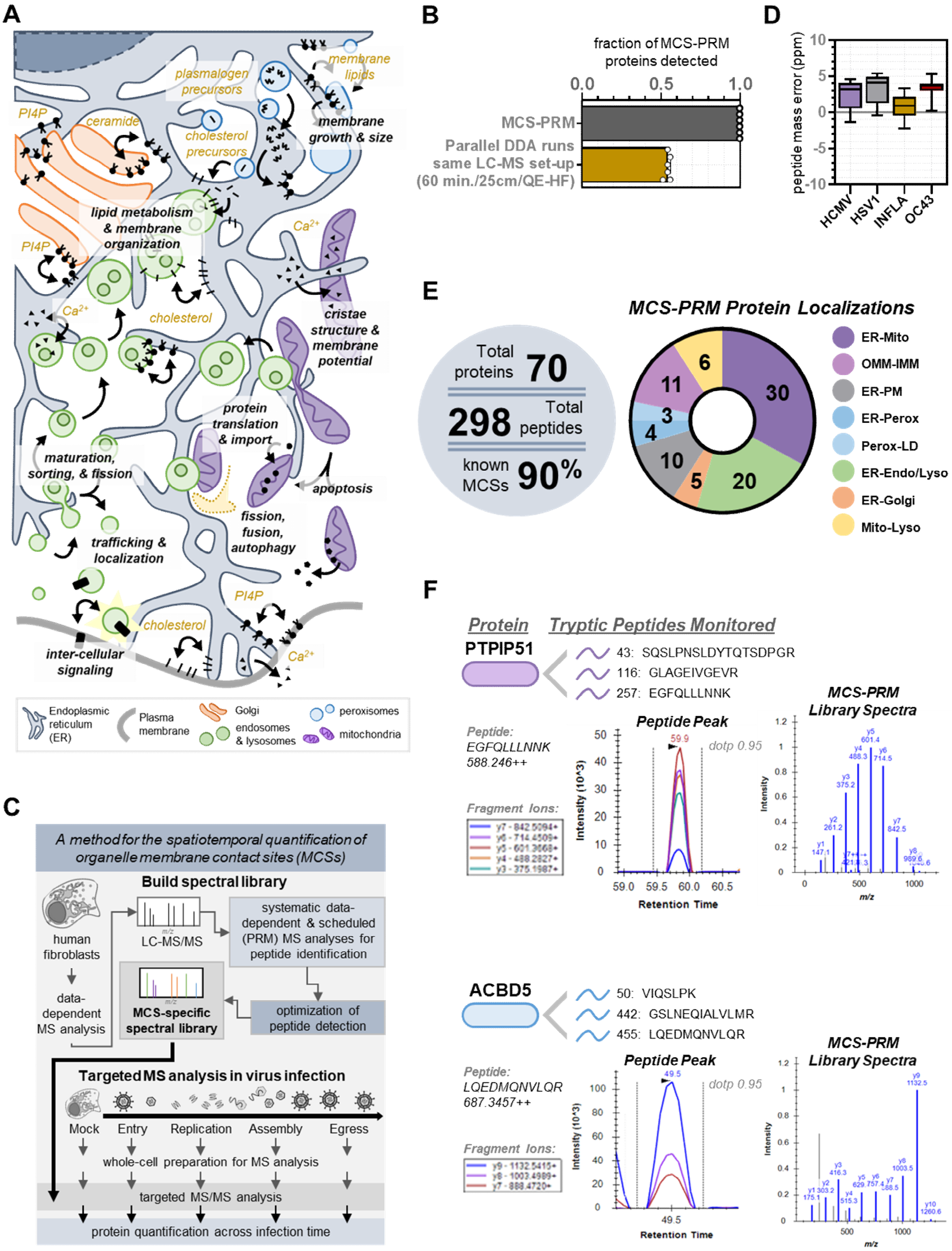

Supplementary Figure 1. *A targeted mass spectrometry assay for simultaneous detection and quantification of MCS proteins (MCS-PRM).*

A. Schematic summarizing the major functions of organelle membrane contact sites (MCSs) known for uninfected cells. Biomolecule transfer functions are in yellow text, and major organelle functions regulated by MCSs are in black.

B. Plot of the fraction of MCS proteins detected in data-dependent (DDA) MS analyses, a common proteomic approach, that were run in parallel to MCS-PRM (N=6 independent samples analyzed separately, indicated by dots, bars represent average % detected across the 6 replicates, error bars are standard deviation). DDA-MS data was acquired using the same instrument (Thermo QE-HF) and liquid chromatography parameters (60 min. gradient, 25 cm column).

C. Development of the MCS-PRM assay first required the identification, development, and validation of a peptide spectral library specific to MCS proteins. For infection studies, samples were collected at timepoints corresponding to major steps in the virus replication cycle (entry, replication, assembly, and egress) and compared to mock/uninfected cells.

D. Box-and-whisker representation of the mass accuracy (ppm) of identified peptides across all biological replicates for each infection in our study (center line is median, whiskers are 10-90 percentiles; including viral peptides N=310 peptides monitored across 6 biological replicates for HCMV, N=293 peptides monitored across 4 biological replicates for HSV-1; N=134 peptides monitored across 3 biological replicates for Infl. A; N=120 peptides monitored across 3 biological replicates for HCoV-OC43). For PRM studies, mass errors are ideally <10 ppm.

E. Report of the proteins and peptides included in our MCS-PRM library (*left*) and donut plot categorizing these proteins by localization (*right*). 90% is an estimated proportion of the functionally-defined MCS proteins reported in peer-reviewed studies, to the best of the authors' knowledge at the time of publication.

F. Examples of MCS-PRM peptides monitored, ion quantification peaks (derived from Skyline[8]), and peptide library spectra for PTPIP51 and ACBD5. *Top*, unique peptides monitored for protein quantification (numbers indicate the amino acid residue at which the peptide begins). *Lower*, representative peptide peak (peptide sequence at *left*) generated in Skyline, plotting MS/MS intensity of fragment ions over LC retention time. Dotted lines indicate window for peak quantification (area under the curve). The dot product (dotp) represents accuracy of experimental peptide peak compared to the library spectra (*right*), which shows each fragment ion for the given peptide labeled with *m/z* and ion number.

See also Supplementary Table 1, Supplementary Data 2.

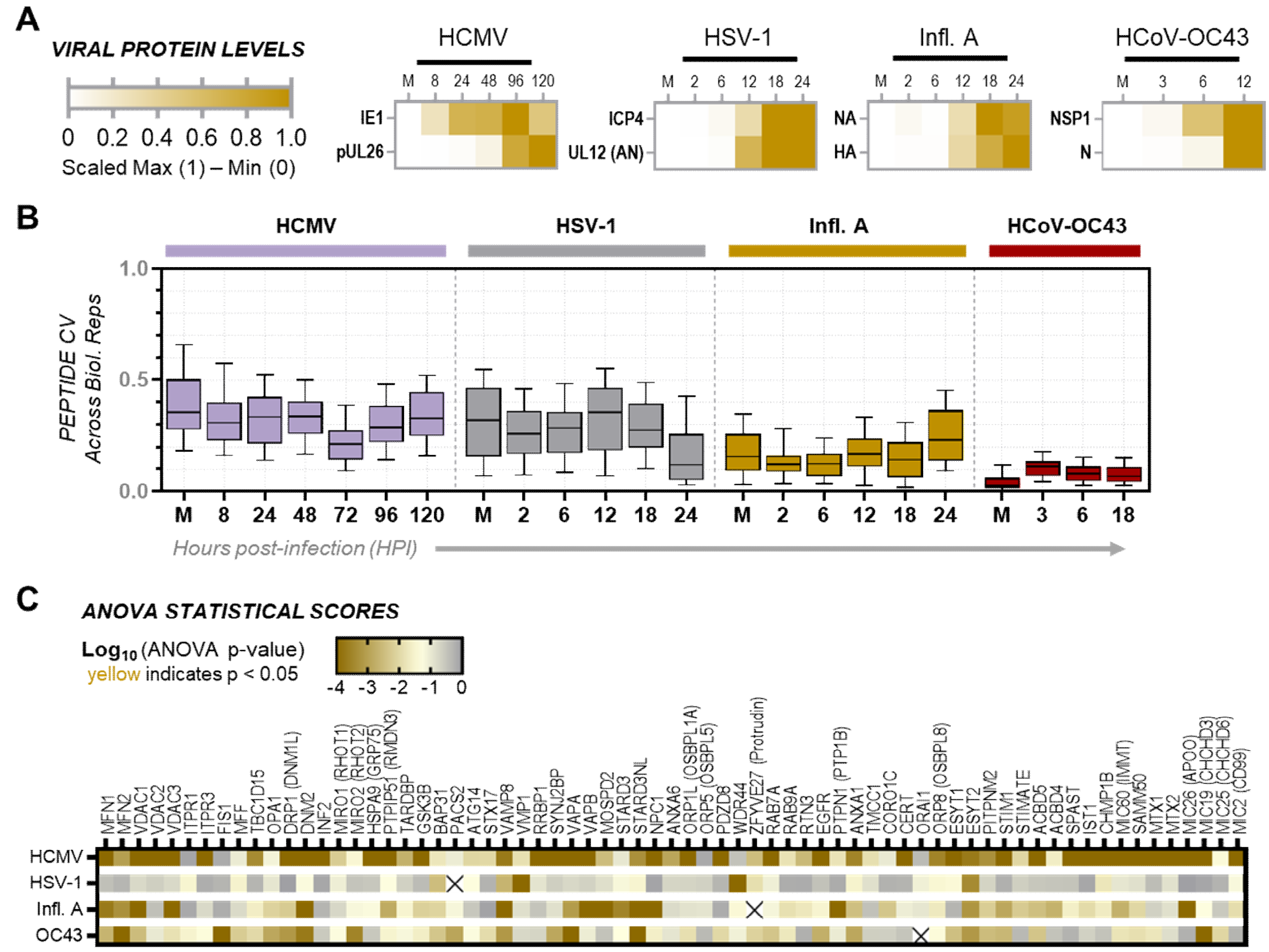

Supplementary Figure 2. *Infection-driven regulation of MCS protein abundances are significant and reproducible.*

A. Analysis of viral protein levels as markers for infection progression, included in the MCS-PRM assay for each infection indicated (all biological replicates). Data is shown as a heatmap scaled by the minimum to maximum abundance values for each protein.

B. Plot of the coefficient of variance (CV) for all peptides monitored by MCS-PRM, calculated for each protein across all biological replicates for the indicated infection (midline is median, whiskers are 10-90 percentiles; including viral peptides N=310 peptides monitored across 6 biological replicates for HCMV, N=293 peptides monitored across 4 biological replicates for HSV-1; N=134 peptides monitored across 3 biological replicates for Infl. A; N=120 peptides monitored across 3 biological replicates for HCoV-OC43).

C. Heatmap depicting one-way ANOVA significance scores for MCS protein abundance changes (from all peptides monitored for each protein across all biological replicates; N=6 biological replicates for HCMV, N=4 for HSV-1, N=3 for Influenza A, N=3 for HCoV-OC43) across the infection with each virus (*top*). P values are colored on a Log_{10} scale, with yellow values representing $P<0.05$ and greyscale values non-significant. Boxes with an 'X' indicate missing values.

See also Figure 1, Supplementary Data 2 for data summaries, and raw data available on the PRIDE and Panorama proteome repositories (check Data Availability section for access information).

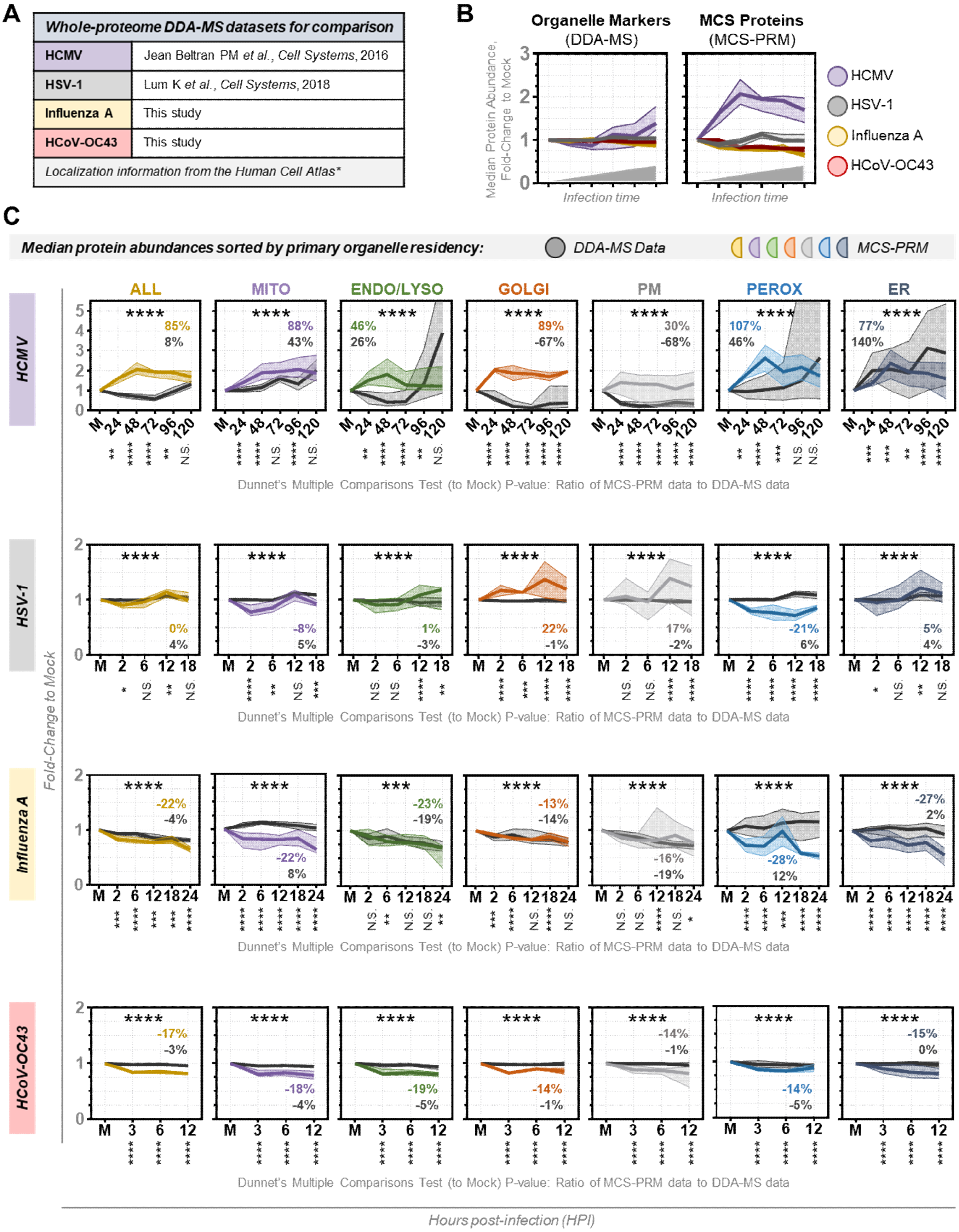

Supplementary Figure 3. *Changes in MCS protein abundances have distinct magnitude and temporality compared to proteins of related localization during virus infections.*

A. Sources of whole-proteome datasets used for comparisons in B-D. Datasets were parsed for high-confidence subcellular localization markers, as determined by the Human Cell Atlas (*Thul *et al.*, 2017[68]). See Supplementary Data 3 for lists of these proteins and their abundance values during infection, as defined by the indicated DDA-MS datasets.

B. Protein abundances during each infection monitored in this study (see color key for infection type at *right*). *Left*, Protein abundance values from the whole-proteome DDA-MS datasets as indicated in A, including all proteins used for the localization comparisons in C and D. *Right,* Protein abundance values from MCS-PRM (this study). Plotted is the median protein abundance at each timepoint as a ratio to uninfected/mock (line, Mock=1), with shaded regions representing the 95% confidence interval.

C. Comparison of MCS-PRM data (colored lines) to abundance changes in whole-proteome DDA-MS data (dark grey lines) for each infection, sorted by localization (derived from the Human Cell Atlas). Plotted is the median protein abundance at each timepoint as a ratio to uninfected/mock (line, Mock/M=1), with shaded regions representing the 95% confidence interval. HCMV values are plotted on a 0-5 scale, while HSV-1, Influenza A, and HCoV-OC43 values are plotted on a 0-2 scale. Asterisks (*) indicate p-values from one-way ANOVA across all timepoints (at the top of each graph) or Dunnet's Multiple Comparisons test to Mock (below each graph) of the ratio of MCS-PRM protein abundances to the DDA-MS marker protein abundances for each category (* $p<0.05$, ** $p<0.01$, *** $p<0.001$, **** $p<0.0001$).

See also Figure 1, Supplementary Data 2 for MCS-PRM data, and Supplementary Data 3 for the localization marker proteins and their corresponding abundance values derived from the DDA-MS datasets. MCS-PRM raw data is available on the PRIDE and Panorama proteome repositories (check Data Availability section for access information).

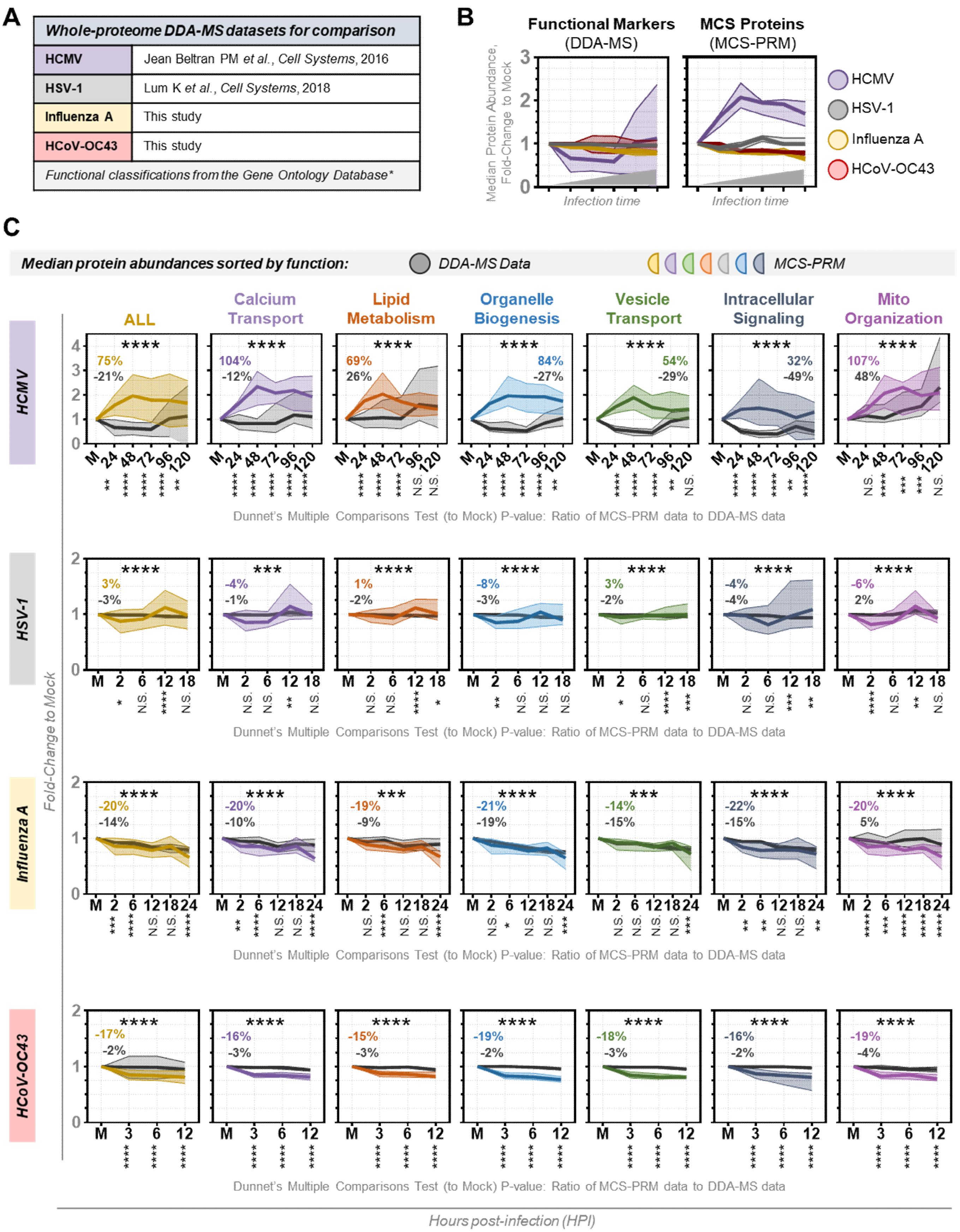

Supplementary Figure 4. *Changes in MCS protein abundances have distinct magnitude and temporality compared to proteins of related function.*

A. Sources of whole-proteome datasets used for comparisons in B-D. Datasets were parsed for gene ontology categories (*The Gene Ontology Consortium[69]) relevant to functions controlled by MCS proteins. See Supplementary Data 3 for lists of these proteins, the gene ontology IDs, and their abundance values during infection, as defined by the indicated DDA-MS datasets.

B. Protein abundances during each infection monitored in this study (see color key for infection type at *right*). *Left*, Protein abundance values from the whole-proteome DDA-MS datasets as indicated in A, including all proteins used for the functional comparisons in C and D. *Right,* Protein abundance values from MCS-PRM (this study). Plotted is the median protein abundance at each timepoint as a ratio to uninfected/mock (line, Mock=1), with shaded regions representing the 95% confidence interval.

C. Comparison of MCS-PRM data (colored lines) to abundance changes in whole-proteome DDA-MS data (dark grey lines) for each infection, sorted by function (derived from the Gene Ontology Database). Plotted is the median protein abundance at each timepoint as a ratio to uninfected/mock (line, Mock/M=1), with shaded regions representing the 95% confidence interval. HCMV values are plotted on a 0-4 scale, while HSV-1, Influenza A, and HCoV-OC43 values are plotted on a 0-2 scale. Asterisks (*) indicate p-values from one-way ANOVA across all timepoints (at the top of each graph) or Dunnett's Multiple Comparisons test to Mock (below each graph) of the ratio of MCS-PRM protein abundances to the DDA-MS marker protein abundances for each category at each timepoint (* $p<0.05$, ** $p<0.01$, *** $p<0.001$, **** $p<0.0001$).

See also Figure 1, Supplementary Data 2 for MCS-PRM data, and Supplementary Data 3 for the functional marker proteins and their corresponding abundance values derived from the DDA-MS datasets. MCS-PRM raw data is available on the PRIDE and Panorama proteome repositories (check Data Availability section for access information).

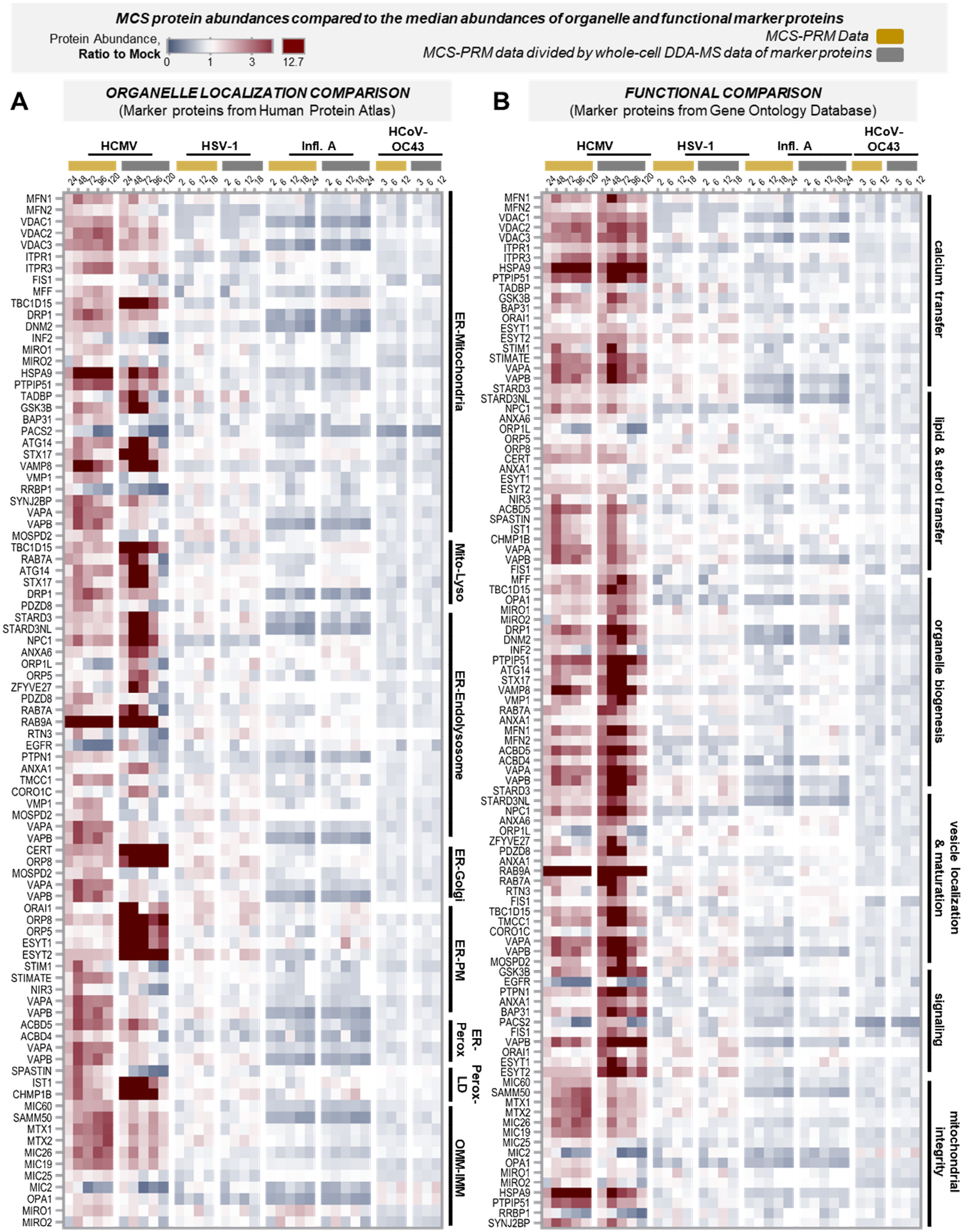

Supplementary Figure 5. *Protein-specific alterations in MCS protein abundances are enriched compared to proteins of related localization or function during virus infections.*

A. Comparison of MCS-PRM data to the abundance values for organelle marker proteins from whole-proteome DDA-MS data, sorted by MCS localization. Data is plotted as a heatmap of protein abundance (Ratio to Mock, key at *top*), with the original MCS-PRM data left (*yellow*) of the given comparison (*grey*), as indicated *above*. Infection time is shown at *top*, protein names are at *left*, and MCS localization is at *right*. Comparisons are to the median protein abundance changes for organelle marker proteins as shown in Supplementary Figure 3. Each protein in MCS-PRM was compared to the median abundance of proteins from its annotated localization. Multi-localized proteins (*e.g.*, ORP5, ORP8 at the plasma membrane and Golgi) were tested for each organelle and thus are shown in each localization category. The MCS-PRM data is already normalized to endogenous peptide controls (from beta-tubulin, histone H2A1A, and histone H2B1A) to control for protein loading and experimental variability.

B. Comparison of MCS-PRM data to the abundance values for functionally similar proteins from whole-proteome DDA-MS data, sorted by MCS functions. Data is plotted as a heatmap of protein abundance (Ratio to Mock, key at *top*), with the original MCS-PRM data left (*yellow*) of the given comparison (*grey*), as indicated *above*. Infection time is shown at *top*, protein names are at *left*, and MCS functions are at *right*. Comparisons are to the median protein abundance changes for functional classes as shown in Supplementary Figure 4. Each protein in MCS-PRM was compared to the median abundance of proteins from its indicated MCS-dependent function. Multi-functional proteins (*e.g.*, VAPs, PTPIP51) were tested for each function and thus are shown in each functional category. The MCS-PRM data is already normalized to endogenous peptide controls (from beta-tubulin, histone H2A1A, and histone H2B1A) to control for protein loading and experimental variability.

See also Figure 1, Supplementary Data 2 for MCS-PRM data, and Supplementary Data 3 for the functional marker proteins and their corresponding abundance values derived from the DDA-MS datasets. MCS-PRM raw data is available on the PRIDE and Panorama proteome repositories (check Data Availability section for access information).

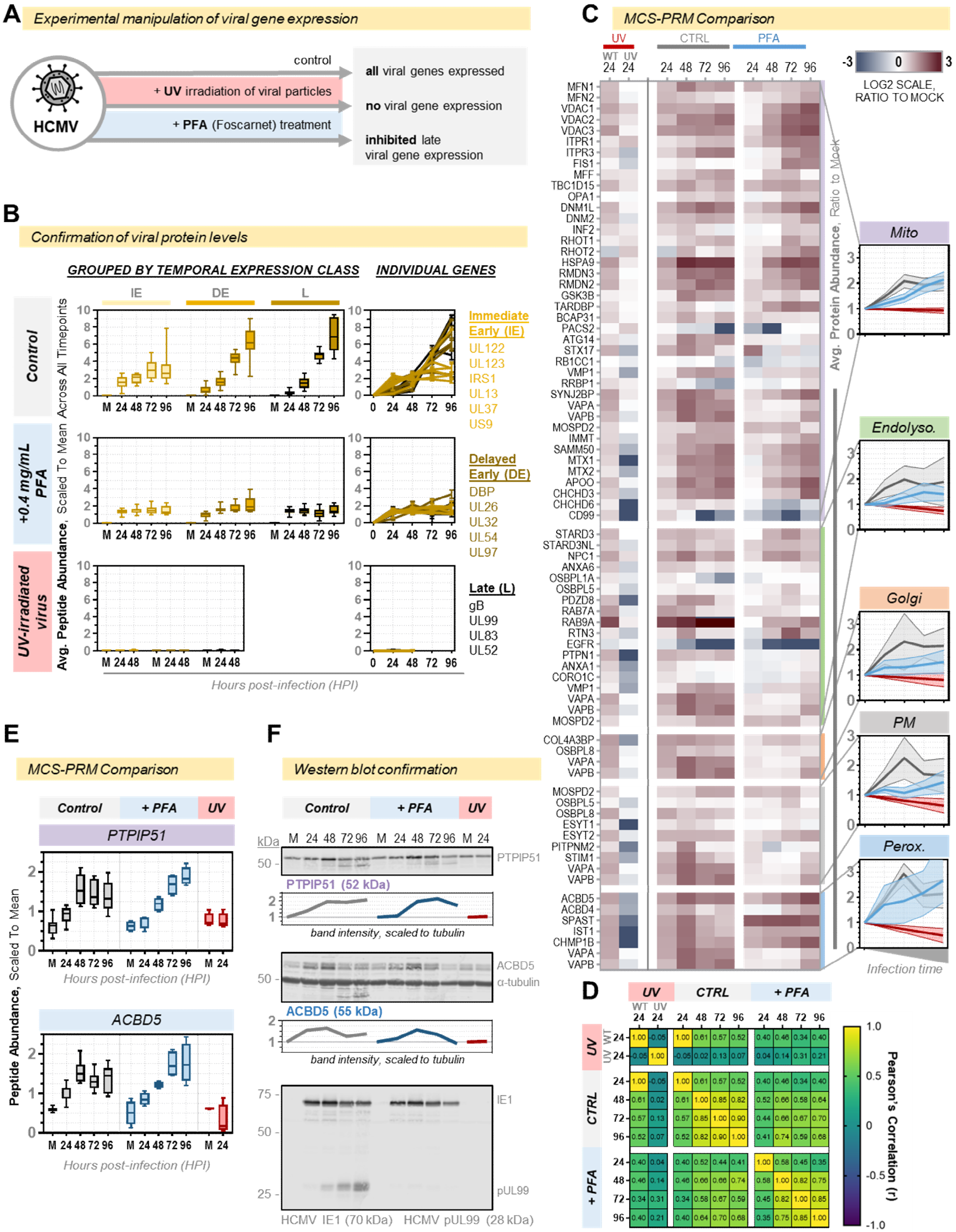
A
Experimental manipulation of viral gene expression
HCMV
control
+ UV irradiation of viral particles
+ PFA (Foscarnet) treatment
all viral genes expressed
no viral gene expression
inhibited late
viral gene expression
B
Confirmation of viral protein levels
GROUPED BY TEMPORAL EXPRESSION CLASS
INDIVIDUAL GENES
IE
DE
L
Control
+0.4 mg/mL PFA
UV-irradiated virus
Avg. Peptide Abundance, Scaled To Mean Across All Timepoints
Immediate Early (IE)
UL122
UL123
IRS1
UL13
UL37
US9
Delayed Early (DE)
DBP
UL26
UL32
UL54
UL97
Late (L)
gB
UL99
UL83
UL52
Hours post-infection (HPI)
C
MCS-PRM Comparison
UV
CTRL
PFA
LOG2 SCALE, RATIO TO MOCK
Avg. Protein Abundance, Ratio to Mock
Mito
Endolyso.
Golgi
PM
Perox.
Infection time
MFN1
MFN2
VDAC1
VDAC2
VDAC3
ITPR1
ITPR3
FIS1
MFF
TBC1D15
OPA1
DNM1L
DNM2
INF2
RHOT1
RHOT2
HSPA9
RMDN3
RMDN2
GSK3B
TARDBP
BCAP31
PACS2
ATG14
STX17
RB1CC1
VMP1
RRBP1
SYNJ2BP
VAPA
VAPB
MOSPD2
IMMT
SAMM50
MTX1
MTX2
APOO
CHCHD3
CHCHD6
CD99
STARD3
STARD3NL
NPC1
ANXA6
OSBPL1A
OSBPL5
PDZD8
RAB7A
RAB9A
RTN3
EGFR
PTPN1
ANXA1
CORO1C
VMP1
VAPA
VAPB
MOSPD2
COL4A3BP
OSBPL8
VAPA
VAPB
MOSPD2
OSBPL5
OSBPL8
ESYT1
ESYT2
PITPNM2
STIM1
VAPA
VAPB
ACBD5
ACBD4
SPAST
IST1
CHMP1B
VAPA
VAPB
D
UV
CTRL
+ PFA
Pearson's Correlation (r)
E
MCS-PRM Comparison
Control
+ PFA
UV
PTPIP51
ACBD5
Peptide Abundance, Scaled To Mean
Hours post-infection (HPI)
F
Western blot confirmation
Control
+ PFA
UV
kDa
PTPIP51
PTPIP51 (52 kDa)
band intensity, scaled to tubulin
ACBD5
α-tubulin
ACBD5 (55 kDa)
band intensity, scaled to tubulin
IE1
pUL99
HCMV IE1 (70 kDa)
HCMV pUL99 (28 kDa)

Supplementary Figure 6. *Viral gene expression is necessary for alterations to MCS protein levels during HCMV infection.*

A. Experimental workflow for determining the contribution of viral genes to the regulation of MCS protein abundances during HCMV infection. UV = ultraviolet light; PFA = phosphonoformic acid, also known as Foscarnet, a clinical drug that prevents viral genome replication and subsequent expression of late (L) viral genes.

B. Viral protein levels across infection time (control, PFA, UV), measured by targeted (PRM) mass spectrometry in parallel with MCS-PRM, confirming infection conditions. *Left*, Box-and-whisker plots of the average protein abundance grouped by each gene class (line at mean, whiskers 10-90 percentiles; N=13 peptides corresponding to 6 proteins measured in 2 independently collected biological replicates; proteins included in each class are listed at *right*). *Right*, Line graphs of individual protein abundances (error bars are standard deviation of peptide measurements; N=2-3 peptides per protein measured in 2 biological replicates), colored by temporal class as listed at *right*.

C. MCS-PRM measurements of MCS proteins in each experimental condition. *Left,* Protein values shown as a heatmap across time (Log2 scale, ratio to mock), sorted by primary protein localization. Conditions and color keys are indicated at *top*, protein names are *left*. Each timepoint is shown as a fold-change to Mock where Mock = 0 (Log2). *Right*, Average changes for MCS proteins at the indicated organelles, comparing control (grey) versus UV (red) and PFA (blue) conditions. Data is shown as a line graph of protein abundances with the line at mean and shaded regions representing the 90-10 confidence intervals. UV values are shown across the entire x-axis to depict the abundance changes on the same scale as the control and PFA conditions.

D. Correlation plot (Pearson's *r* values) comparing protein abundances in control, UV, are PFA samples. Color key is *below*, with yellow representing high correlation and blue representing low correlation.

E. Box-and-whisker plots showing PTPIP51 and ACBD5 peptide abundances as defined by MCS-PRM in C. Shown as average peptide abundance as a ratio to mean, with line at mean and whiskers 10-90 percentiles (N=8 peptide measurements in 2 independently collected biological replicates for PTPIP51, N=11 peptide measurements in 2 independently collected biological replicates for ACBD5). Each condition is indicated at *top*.

F. Western blot analysis of PTPIP51 and ACBD5 abundance changes in control, UV, and PFA conditions. Timepoints/conditions are indicated at *top* (M = Mock, numbers represent timepoints in hours post-infection). The densitometry quantification of band intensities is shown beneath each blot (all densitometry data is scaled to tubulin as a loading control). HCMV viral proteins IE1 (immediate early class) and pUL99 (late class) are used to confirm infection conditions. Samples used for western blotting are an additional biological replicate than those used for MCS-PRM (N=2 biological replicates for MCS-PRM in panels C-E), and western blotting was one biological replicate.

See also Figure 1.

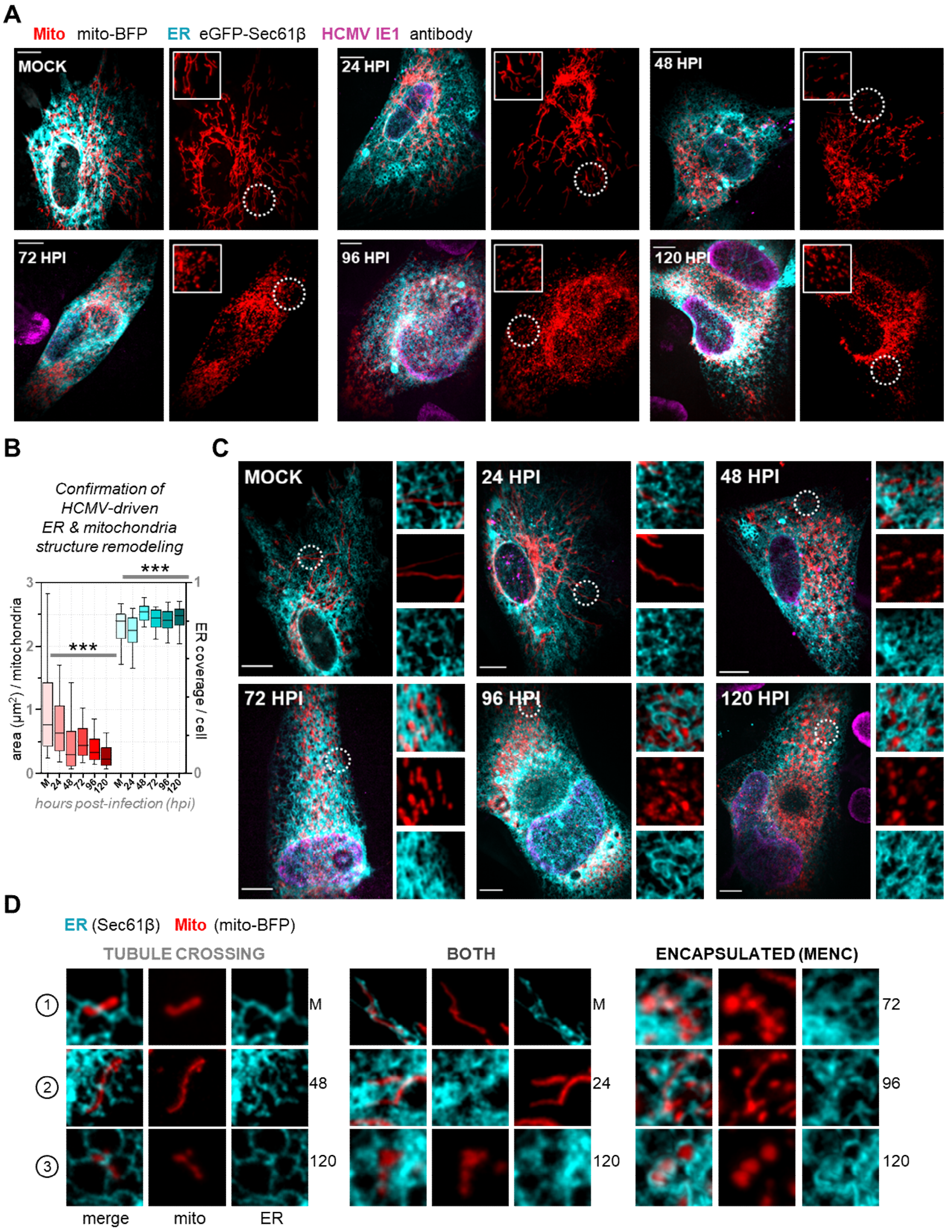
A
Mito mito-BFP ER eGFP-Sec61β HCMV IE1 antibody
MOCK
24 HPI
48 HPI
72 HPI
96 HPI
120 HPI
B
Confirmation of HCMV-driven ER & mitochondria structure remodeling
***
***
area (μm²) / mitochondria
ER coverage / cell
M 24 48 72 96 120 M 24 48 72 96 120
hours post-infection (hpi)
C
MOCK
24 HPI
48 HPI
72 HPI
96 HPI
120 HPI
D
ER (Sec61β) Mito (mito-BFP)
TUBULE CROSSING
BOTH
ENCAPSULATED (MENC)
1
2
3
M
48
120
M
24
120
72
96
120
merge
mito
ER

Supplementary Figure 7. *HCMV infection alters ER-mitochondria morphologies and interactions into stable MENCs.*

A. Maximum projections of immunofluorescent images of fibroblasts infected with HCMV and labeled for ER (cyan), mitochondria (red), and the viral protein IE1 (magenta). A 7x7µm ROI from the mitochondrial channel for each timepoint is shown in a white-bordered box. Scale bars (above hpi labels) represent 10µm. Images are representative of many (N>20) independent experiments that obtained similar results.

B. Quantification of mitochondrial size (*left y-axis*) and ER density (*right y-axis*) from images in A, shown as box-and-whisker plots with midline representing the mean and whiskers Tukey distribution (N=36, 25, 25, 28, 34, 43 cells per Mock, 24, 48, 72, 96, 120 hpi, respectively; ***p≤0.0001 by one-way ANOVA with Dunnett's multiple comparisons test to Mock).

C. Immunofluorescent images of cells as in A, highlighting ER-mitochondria interactions as HCMV infection progresses. These images are representative of each timepoint for the quantification performed in Figure 2D. Scale bars 10µm. Images are representative of many (N>20) independent experiments that obtained similar results.

D. Zoomed regions of ER-mitochondria interaction phenotypes observed across HCMV infection time, from cells as in A and C. Each image is a 7x7µm region, and timepoints are indicated at *right*, depicting examples from every timepoint that are representative of the phenotypes observed regardless of mitochondrial size. Images are representative of many (N>20) independent experiments that obtained similar results.

See also Figure 2, Supplementary Movies 1-2.

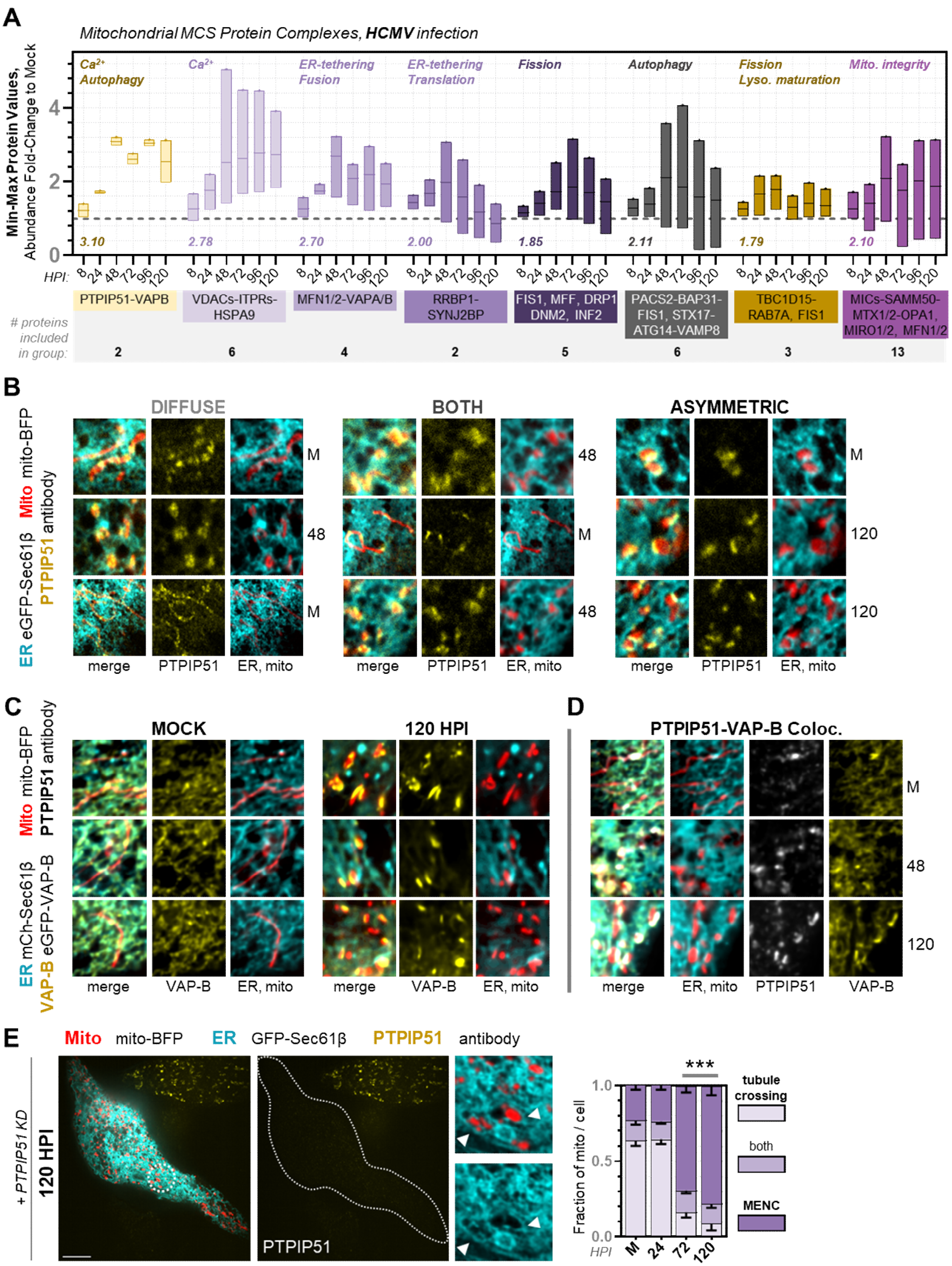

A
Mitochondrial MCS Protein Complexes, HCMV infection
Min-Max Protein Values, Abundance Fold-Change to Mock
Ca2+ Autophagy
Ca2+
ER-tethering Fusion
ER-tethering Translation
Fission
Autophagy
Fission Lyso. maturation
Mito. integrity
3.10
2.78
2.70
2.00
1.85
2.11
1.79
2.10
HPI:
PTPIP51-VAPB
VDACs-ITPRs-HSPA9
MFN1/2-VAPA/B
RRBP1-SYNJ2BP
FIS1, MFF, DRP1 DNM2, INF2
PACS2-BAP31-FIS1, STX17-ATG14-VAMP8
TBC1D15-RAB7A, FIS1
MICs-SAMM50-MTX1/2-OPA1, MIRO1/2, MFN1/2
proteins included in group:
2
6
4
2
5
6
3
13
B
DIFFUSE
BOTH
ASYMMETRIC
ER eGFP-Sec61β Mito mito-BFP PTPIP51 antibody
merge
PTPIP51
ER, mito
C
MOCK
120 HPI
ER mCh-Sec61β Mito mito-BFP VAP-B eGFP-VAP-B PTPIP51 antibody
VAP-B
D
PTPIP51-VAP-B Coloc.
E
Mito mito-BFP ER GFP-Sec61β PTPIP51 antibody
+ PTPIP51 KD
120 HPI
PTPIP51
Fraction of mito / cell
***
tubule crossing
both
MENC

Supplementary Figure 8. *PTPIP51 and VAP-B accumulate at HCMV-driven ER-mitochondria encapsulations.*

A. Protein abundances of mitochondrial MCS proteins during HCMV infection, categorized by known interactions into MCS complexes *(below* in colored boxes), as referenced in Table S1. Plotted is the minimum to maximum fold-change (to Mock = 1) of MCS-PRM data for the proteins in each category across time (hpi on x-axis), with lines representing the mean (box bounds are the minima and maxima for the protein abundances for each complex). The primary functions of each MCS is at *top*, and the maximum average fold-change across all timepoints is indicated in the numbers below each graph (values here are average protein abundance across all proteins assigned to the complex, N=6 biological replicates as quantified by MCS-PRM during HCMV infection).

B. Representative images of endogenous PTPIP51 localization at ER-mitochondria junctions as HCMV infection progresses. Timepoints for each region of interest (taken from images as in Figure 3) is indicated at *right*. Images are representative of many (N>5) independent experiments that obtained similar results.

C. Representative images (7x7µm) of live cells showing VAP-B localization at ER-mitochondria junctions prior to (Mock) and after infection (120 hpi). Images are representative of N=3 independent experiments that obtained similar results and are quantified in Figure 3.

D. Representative images (7x7µm) of fixed cells labeled for endogegnous PTPIP51 and VAP-B, showing co-accumulation at ER-mitochondria junctions as HCMV infection progresses. Timepoints are indicated at *right*. Images are representative of N=3 independent experiments that obtained similar results.

E. Representative image (confocal z-slice) of PTPIP51 KD cells at 120 hpi, labeled for ER (cyan), mitochondria (red), and PTPIP51 (yellow). Zooms (white circle, at *right*) show mito-ER encapsulations (MENCs, white arrows) present in the absence of PTPIP51 (cell outline defined in white in PTPIP51 channel). Quantification is shown at *right*, scoring mitochondria-ER interaction phenotypes (N=20, 20, 19, 10 cells for Mock, 24, 72, 120 hpi, respectively; ≥17 mitochondria/cell; error bars are SEM, ***p≤0.0001 by two-way ANOVA with Dunnett's multiple comparisons test to Mock).

See also Figure 3.

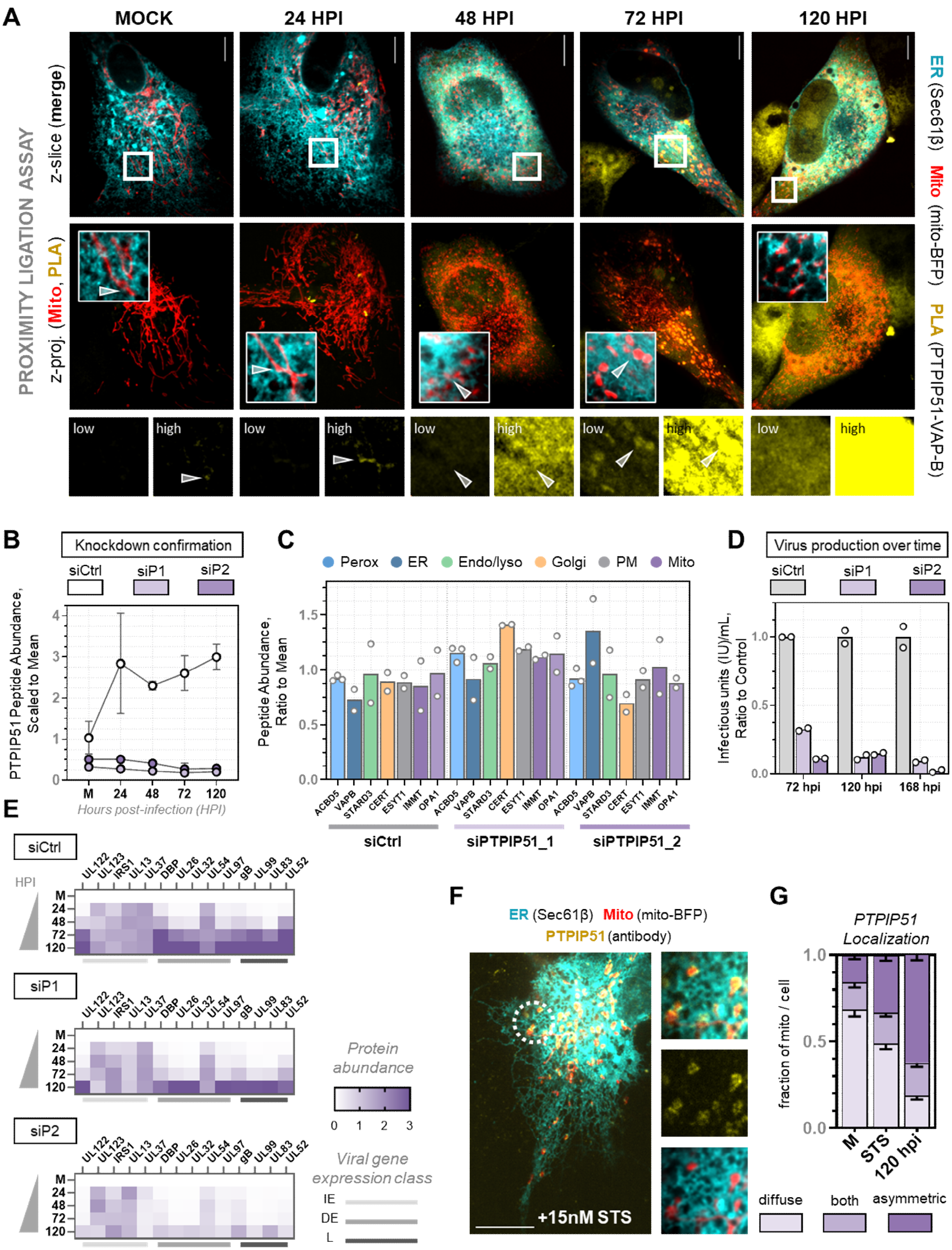
A
MOCK
24 HPI
48 HPI
72 HPI
120 HPI
PROXIMITY LIGATION ASSAY
z-slice (merge)
z-proj. (Mito, PLA)
low
high
ER (Sec61β) Mito (mito-BFP) PLA (PTPIP51-VAP-B)
B
Knockdown confirmation
siCtrl siP1 siP2
PTPIP51 Peptide Abundance, Scaled to Mean
Hours post-infection (HPI)
C
Perox ER Endo/lyso Golgi PM Mito
Peptide Abundance, Ratio to Mean
ACBD5 VAPB STARD3 CERT ESYT1 IMMT OPA1
siCtrl
siPTPIP51_1
siPTPIP51_2
D
Virus production over time
siCtrl siP1 siP2
Infectious units (IU)/mL, Ratio to Control
72 hpi
120 hpi
168 hpi
E
siCtrl
siP1
siP2
HPI
UL122 UL123 IRS1 UL13 UL37 DBP UL26 UL32 UL54 UL97 gB UL99 UL83 UL52
Protein abundance
Viral gene expression class
IE
DE
L
F
ER (Sec61β) Mito (mito-BFP)
PTPIP51 (antibody)
+15nM STS
G
PTPIP51 Localization
fraction of mito / cell
M
STS
120 hpi
diffuse
both
asymmetric

Supplementary Figure 9. *PTPIP51 increases in VAP-B tethering interactions and is required for HCMV production.*

- **A.** Images from proximity ligation assay (PLA) of endogenous PTPIP51-VAP-B interactions across HCMV infection time. Channels from a zoomed region (indicated by a white box) are shown *below*, including both low and high exposures of PLA signal (yellow) to highlight the progression of PTPIP51-VAP-B interactions. The same exposure settings were used for each image. Arrows indicate PLA puncta associated with ER-mitochondria junctions. See quantification in Figure 3. Scale bars 10µm.
- **B.** Quantification of PTPIP51 abundance before and during HCMV infection, as determined by MCS-PRM, in siRNA control (siCtrl; white) versus PTPIP51 KD cells (two siRNAs indicated as siP1 and siP2; purple). Plotted is the average peptide abundance from three peptides quantified at each timepoint (indicated on x-axis in hpi, M=Mock), scaled to the mean peptide abundance across all conditions (N=6 total peptide values for each timepoint, from 3 peptides measured in 2 independently collected biological replicates). Error bars are standard deviation.
- **C.** Protein levels of MCS proteins localized to other MCSs upon siRNA knockdowns in uninfected cells, comparing siRNA control (siCtrl) to PTPIP51 KDs (siP1, siP2). Data is shown as average peptide abundance, with error bars standard error of the mean, measured by MCS-PRM (N=2 biological replicates, each dot represents an average peptide value across replicates). Localization key is at *top* ("mito" = outer-inner mitochondrial membrane contacts; "IMMT" also known as Mic60).
- **D.** HCMV titers measured at 72, 120, and 168 hours post-infection in control (grey) versus PTPIP51 KD (purple) cells. Shown as a ratio to control for each timepoint (N=2 biological replicates).
- **E.** Targeted MS quantification of viral protein abundances (N=2 biological replicates) in control versus PTPIP51 KD cells, from the temporal expression classes of immediate early (IE), delayed early (DE), and late (L), as indicated in the greyscale key at right.
- **F.** Staurosporine (STS)-treated human fibroblast cell (15nM STS for 30 min.) with fragmented mitochondria labeled for ER (cyan), mitochondria (red), and endogenous PTPIP51 (yellow). Two ROIs are shown, indicated by red and yellow circles. Scale bar is 10µm.
- **G.** Quantification of images as in D, scoring PTPIP51 accumulation at ER-mitochondria junctions in mock versus STS-treated or HCMV 120 hpi cells (scored for ≥15 mitochondria/cell for N=17 cells in Mock, N=20 cells in STS and 120 hpi corresponding to two independent experiments; error bars are SEM).

See also Figure 3.

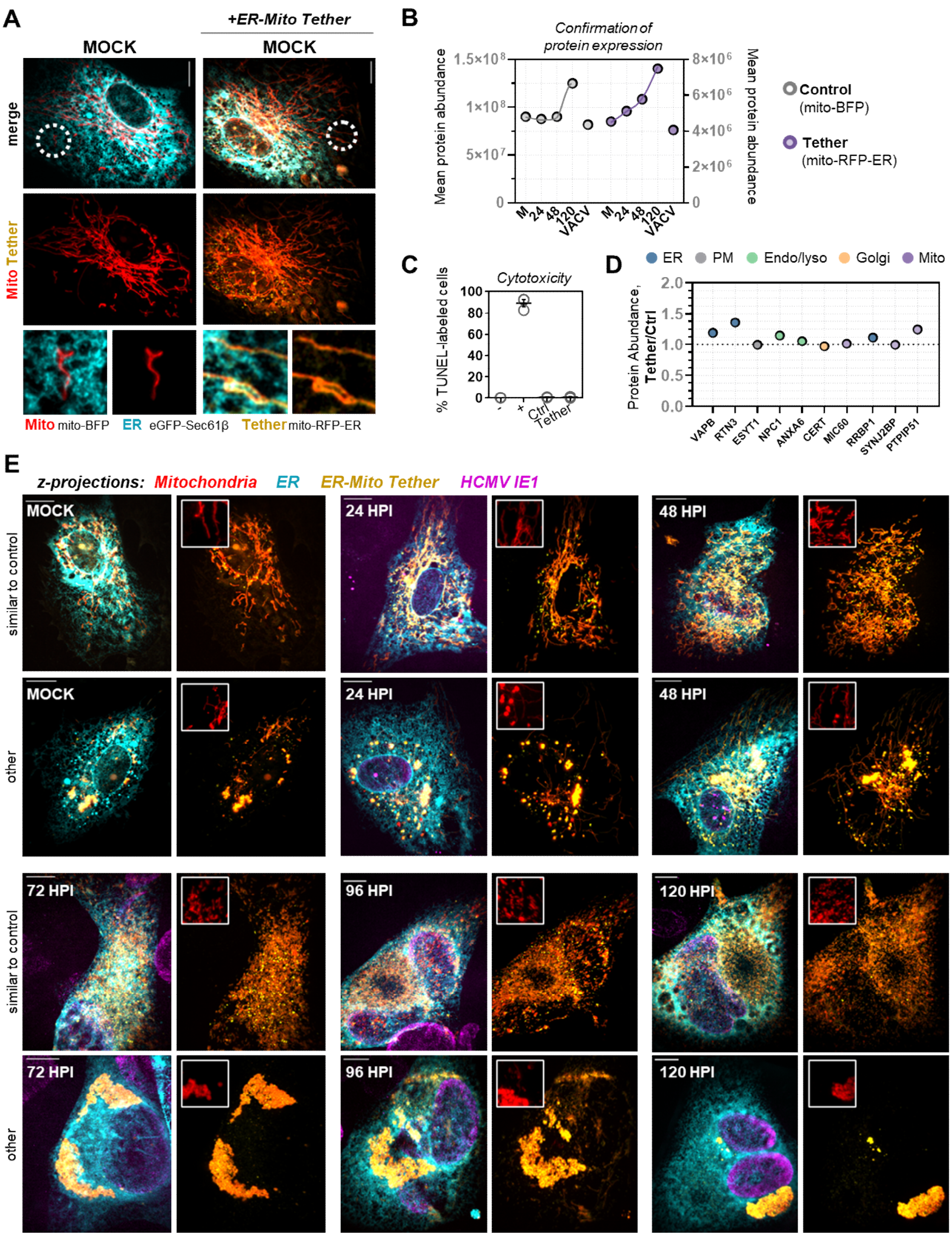

A
+ER-Mito Tether
MOCK
MOCK
merge
Mito Tether
Mito mito-BFP
ER eGFP-Sec61β
Tether mito-RFP-ER
B
Confirmation of protein expression
Mean protein abundance
1.5×10^8
1×10^8
5×10^7
0
8×10^6
6×10^6
4×10^6
2×10^6
0
M
24
48
120
VACV
Control (mito-BFP)
Tether (mito-RFP-ER)
C
Cytotoxicity
% TUNEL-labeled cells
100
80
60
40
20
0
-
+
Ctrl
Tether
D
ER
PM
Endo/lyso
Golgi
Mito
Protein Abundance, Tether/Ctrl
2.0
1.5
1.0
0.5
0.0
VAPB
RTN3
ESYT1
NPC1
ANXA6
CERT
MIC60
RRBP1
SYNJ2BP
PTPIP51
E
z-projections: Mitochondria ER ER-Mito Tether HCMV IE1
similar to control
other
MOCK
24 HPI
48 HPI
72 HPI
96 HPI
120 HPI

Supplementary Figure 10. *Synthetic ER-mitochondria tethering disrupts mitochondrial dynamics during HCMV infection.*

A. Comparison of cells without (*left*) and with (*right*) expression of the mito-RFP-ER synthetic tether (yellow). Scale bars 10μm. Images are representative of many (N>8) independent experiments that obtained similar results.

B. DDA-MS quantification of control (grey, mito-BFP, *left* y-axis) and tether (mito-RFP-ER, purple, *right* y-axis) expression through 120 hours post-HCMV infection and 6 hours post-VACV 70mer (vaccinia virus DNA) transfection (shown are the values from one biological replicate, with N=17 and 7 unique peptide quantifications for mito-BFP and mito-RFP-ER, respectively, as quantified by Proteome Discoverer; data is representative of 5 independent experiments; line connects the mean protein abundances to exhibit maintenance of protein expression over HCMV infection time).

C. TUNEL assay measurement of cytoxicity in control (Ctrl) versus mito-RFP-ER (Tether) cells, including assay positive (+) and negative (-) controls (N=3 biological replicates).

D. Quantification of MCS proteins (DDA-MS) from different subcellular locations upon mito-RFP-ER tether expression in uninfected cells, plotted as the ratio of tether/control (N=6, 4, 30, 5, 68, 3, 28, 77, 2, 2 peptides quantified across two independently collected biological replicates for VAP-B, RTN3, ESYT1, NPC1, ANXA6, CERT, MIC60, RRBP1, SYNJ2BP, PTPIP51, respectively, as quantified by Proteome Discoverer; line at y=1 represents no change between tether and control abundances).

E. Immunofluorescent images of fibroblasts transfected with the ER-mitochondria tether (mito-RFP-ER, yellow), labeled for ER (eGFP-Sec61β, cyan) and mitochondria (mito-BFP, red), and stained for the viral protein IE1 (magenta) to confirm infection. For each timepoint, an example of "control-like" mitochondria phenotypes are shown above an example of aberrant mitochondrial dynamics (reference Supplementary Figure 6 for control comparisons). Scale bars (above timepoint labels) represent 10μm. Images are representative of three independent experiments that obtained similar results

See also Figure 4.

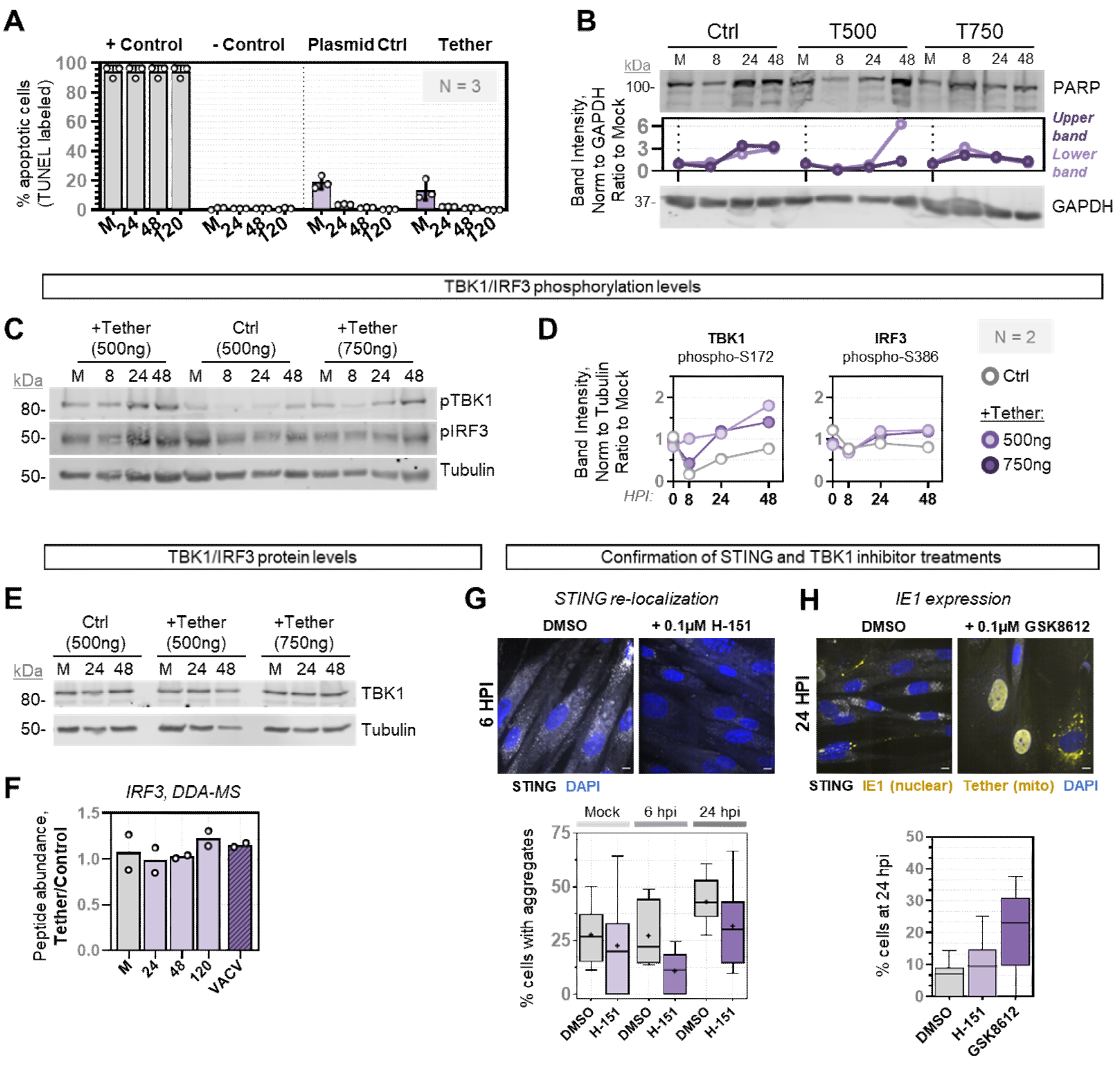

Supplementary Figure 11. *Premature ER-mitochondria tethering promotes STING-TBK1-IRF3 activation.*

A. Quantification of apoptotic cell populations, as determined by TUNEL assay, in control versus ER-mitochondria tether (500ng) cells infected with HCMV. Timepoints are indicated at bottom of graph, error bars represent standard error of the mean (N=3 biological replicates).

B. Western blot analysis of PARP cleavage upon HCMV infection in cells transfected with mito-RFP-ER tether (T500=500ng, T750=750ng) compared to control. Line graph depicts the densitometry quantification of PARP bands normalized to GAPDH and plotted as a ratio to the mock/uninfected timepoint for each condition. Image is representative of two independent experiments.

C. Western blot of TBK1/IRF3 phosphorylation in control versus mito-ER tether cells during HCMV infection (timepoints at *top*), with endogenous tubulin as a loading control. Image is representative of two independent experiments. See Source Data for uncropped blot.

D. Densitometry of pTBK1 and pIRF3 bands from C, scaled to tubulin and shown as a ratio to mock (N=2 biological replicates, dot is at mean and bars represent the full range of the two independent replicates).

E. Western blot of TBK1 protein levels during HCMV infection (timepoints at *top*) in control versus mito-ER tether cells, with endogenous tubulin as a loading control. Image is representative of two independent experiments. See Source Data for uncropped blot.

F. Targeted MS quantification of IRF3 protein levels during HCMV infection and VACV 70mer DNA transfection. Plotted is the average IRF3 peptide abundance, error bars are standard deviation (N=2 biological replicates).

G. Quantification of STING re-localization into cytoplasmic aggregates upon treatment with the STING inhibitor H-151. *Top*, example images of fibroblasts transfected with the mito-RFP-ER tether, treated with H-151, and stained for STING (grey) and DAPI (blue), at 6 hpi with HCMV. *Lower*, Quantification of tether cells before infection and at 6 and 24 hpi, imaged as above, represented as a box-and-whisker plot (line at median, whiskers are 10-90 percentile, + at mean; N=142 cells for Mock DMSO, N=114 for Mock H-151, N=121 for 6 hpi DMSO, N=113 for 6 hpi H-151, N=159 for 24 hpi DMSO, N=153 for 24 hpi H-151, altogether corresponding to two independently acquired microscopy experiments). DMSO treatment was used as a control for drug treatment.

H. Quantification of detectable IE1 by immunofluorescence staining at 24 hours post-HCMV infection in tether-expressing cells, indicating HCMV infection onset. Compared are DMSO-treated cells to cells treated with STING (H-151) and TBK1 (GSK8612) inhibitors (data is plotted as a box-and-whisker plot where midline is at median and whiskers are Tukey distribution; N=159 cells for DMSO, N=153 cells for H-151, N=140 cells for GSK8612, altogether corresponding to two independently acquired microscopy experiments).

See also Figure 4.

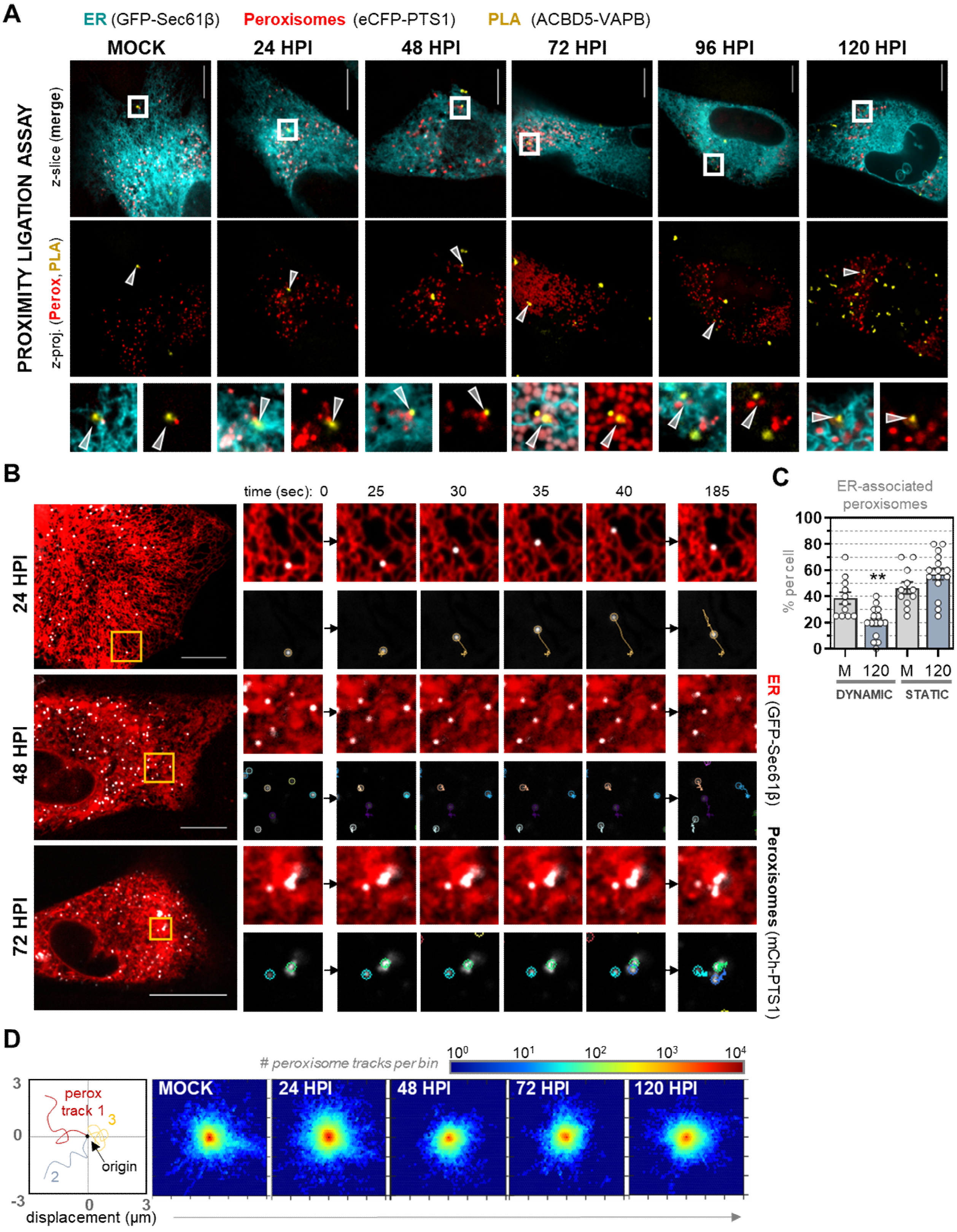

Supplementary Figure 12. *ER-peroxisome and ACBD5-VAPB tethering interactions are increased by HCMV infection.*

A. Images from proximity ligation assay (PLA) of endogenous ACBD5-VAP-B interactions across HCMV infection time, confirming PLA (yellow) localization near ER (cyan) and peroxisomes (red). Arrows indicate PLA puncta associated with ER-peroxisome junctions. Quantification is shown in Figure 5 (N≥40 cells per timepoint).

B. Representative images from live-cell analysis of ER-peroxisome interactions during HCMV infection of timepoints not shown in the main figure (Fig. 5). A region of interest from each cell is shown at right as 5 second interval shots, including examples of the peroxisome tracks used for motility analysis shown in C. Scale bars represent 10μm. Images are representative of many (N>10) independent experiments that obtained similar results, and are quantified in panel D and in Figure 5.

C. Quantification of ER-associated peroxisome dynamics, plotting the fraction per cell displaying dynamic versus static movement (N=11 cells for Mock, N=15 cells for 120 hpi, **p=0.0017 by two-tailed student's t-test of Dynamic Mock versus 120 hpi).

D. Particle-tracking of peroxisomes, plotted in 2D histograms (binned by x-y displacement of individual tracks from 3 min. movies, N≥1500 peroxisomes, N≥11 cells/timepoint). Heat-colored key is above.

See also Figure 5, Supplementary Movie 3.

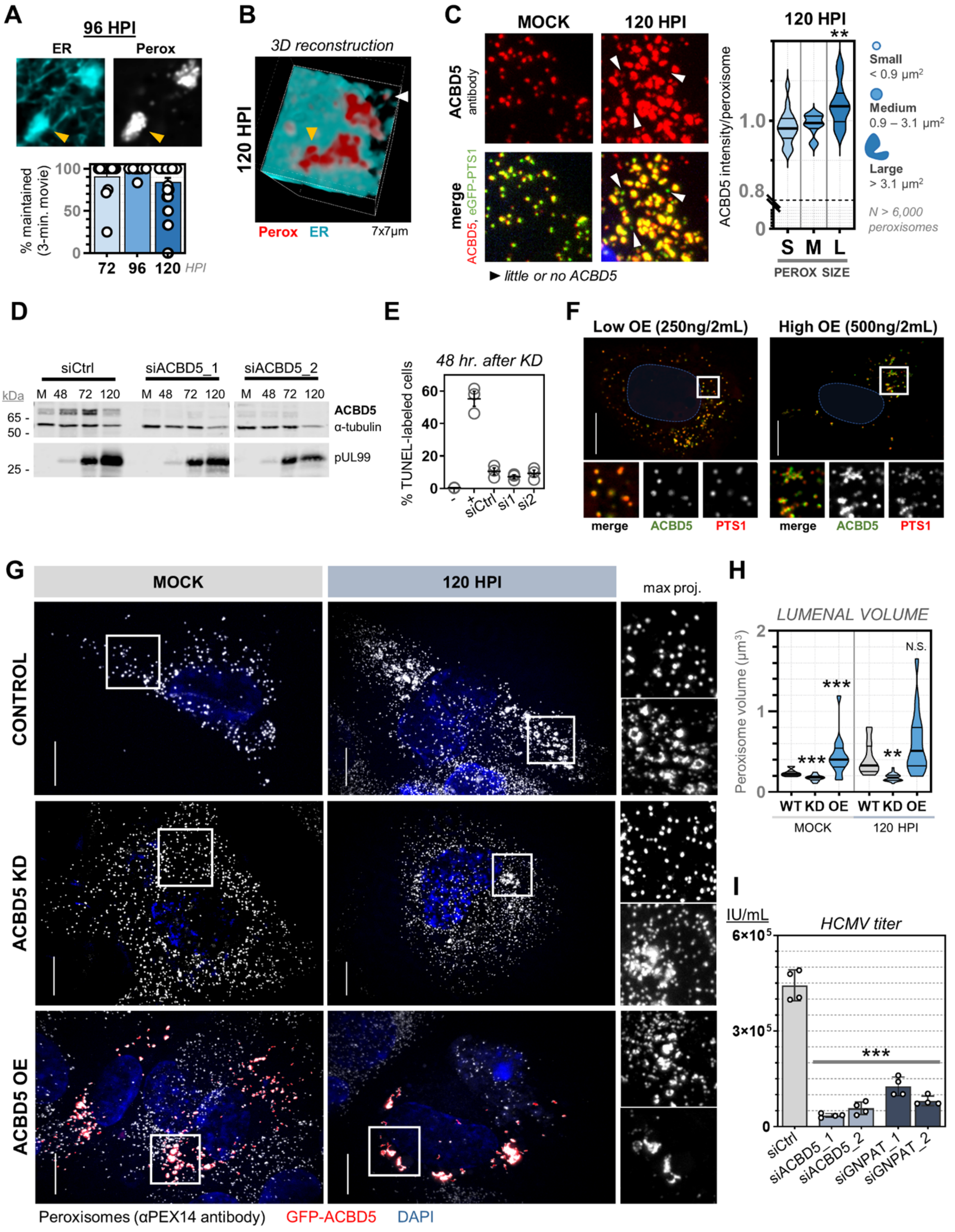

A
96 HPI
ER
Perox
% maintained (3-min. movie)
100
50
0
72
96
120
HPI
B
3D reconstruction
120 HPI
Perox
ER
7x7µm
C
MOCK
120 HPI
ACBD5 antibody
merge
ACBD5, eGFP-PTS1
► little or no ACBD5
120 HPI
**
ACBD5 intensity/peroxisome
1.0
0.8
0
S
M
L
PEROX SIZE
Small
< 0.9 µm²
Medium
0.9 – 3.1 µm²
Large
> 3.1 µm²
N > 6,000 peroxisomes
D
siCtrl
siACBD5_1
siACBD5_2
kDa
M 48 72 120
65 -
50 -
25 -
ACBD5
α-tubulin
pUL99
E
48 hr. after KD
% TUNEL-labeled cells
60
40
20
0
-
+
siCtrl
si1
si2
F
Low OE (250ng/2mL)
High OE (500ng/2mL)
merge
ACBD5
PTS1
G
MOCK
120 HPI
max proj.
CONTROL
ACBD5 KD
ACBD5 OE
Peroxisomes (αPEX14 antibody)
GFP-ACBD5
DAPI
H
LUMENAL VOLUME
Peroxisome volume (µm³)
2
1
0
***
***
**
N.S.
WT KD OE
WT KD OE
MOCK
120 HPI
I
IU/mL
HCMV titer
6×10⁵
3×10⁵
0
***
siCtrl
siACBD5_1
siACBD5_2
siGNPAT_1
siGNPAT_2

Supplementary Figure 13. *ER-peroxisome contacts are enriched at virus-driven enlarged peroxisomes and required for their formation.*

A. Enlarged peroxisomes, which begin forming at 72 hpi, maintain association with expanded areas of ER, quantified from 3 min. movies (bars represent mean, each dot is a cell value tracking all enlarged peroxisomes for the movie duration, N≥15 cells/timepoint).

B. 3D reconstruction of a zoom from a 120 hpi cell labelled for ER (cyan, eGFP-Sec61β) and peroxisome membranes (red, PEX14 antibody), as in Figure 5. Arrows indicate enlarged (yellow) or small (white) peroxisomes associated with expanded ER or ER tubules, respectively.

C. *Left*, Representative 10x10μm regions from an immunofluorescent analysis of endogenous ACBD5 localization before and 120 hpi. Shown are maximum projections of z-stack images, with peroxisomes (green, eGFP-PTS1) and ACBD5 (red, antibody). Signal overlap is in yellow. Arrows indicate peroxisomes lacking ACBD5 at 120 hpi, which are of the fragmented population. *Right*, Quantification of ACBD5 co-localization with individual peroxisomes categorized by surface area size (categories shown at right, N≥6,000 peroxisomes, N≥9 cells per timepoint, **p=0.002 by two-tailed student's t-test of L versus S).

D. Western blot confirming ACBD5 KDs across 120 hours of HCMV infection (tubulin is loading control, pUL99 is an HCMV protein controlling for infection progression). Image is representative of three independent experiments that obtained similar results. See Source Data for uncropped blot.

E. Cytotoxicity, as determined by TUNEL assay of apoptotic cells, of ACBD5 siRNA-mediated KDs is comparable to control (siCtrl) (N=3 biological replicates, all points are shown, line is at mean and error bars are SD). Assay also includes positive (+) and negative (-) controls.

F. Confocal microscopy of cells labeled with eGFP-ACBD5 (green) and mCh-PTS1 (red), confirming ACBD5 expression and localization to peroxisome membranes after transfection with two amounts of plasmid (250ng, *left* and 500ng, *right*). Images are representative of many (N>5) independent experiments that obtained similar results.

G. Additional immunofluorescent images of peroxisomes (white) in control versus ACBD5 KD and OE cells before infection and at 120 hpi, with zoomed regions (shown *right*) highlighting differences in peroxisome numbers and morphology. These complement examples given in Figure 5. Scale bars represent 10μm. Images are representative of two independent experiments that obtained similar results, and are quantified in panel H and in Figure 5.

H. Quantification of peroxisome volume in control (WT, N=15 cells/timepoint), ACBD5 KD (N=15 Mock and N=28 infected cells/timepoint), and ACBD5 OE (N=15 cells/timepoint) cells before and 120 hpi (≥4,000 peroxisomes quantified per condition; **p≤0.01, ***p≤0.001 by two-tailed student's t-test to WT for each timepoint).

I. Virus titer measurements from cells with siRNA-mediated KDs of ACBD5 and GNPAT, using two siRNAs per gene (N=4 biological replicates, error bars are SD, ***p≤0.0001 by two-tailed student's t-test to siCtrl).

See also Figure 5, Supplementary Movies 4-5.

SUPPLEMENTARY REFERENCES

1. De Brito, O. M. & Scorrano, L. Mitofusin 2 tethers endoplasmic reticulum to mitochondria. *Nature* **456**, 605–610 (2008).
2. Abrisch, R. G., Gumbin, S. C., Wisniewski, B. T., Lackner, L. L. & Voeltz, G. K. Fission and fusion machineries converge at ER contact sites to regulate mitochondrial morphology. *J. Cell Biol.* **219**, (2020).
3. Szabadkai, G. *et al.* Chaperone-mediated coupling of endoplasmic reticulum and mitochondrial Ca2+ channels. *J. Cell Biol.* **175**, 901–911 (2006).
4. Hung, V. *et al.* Proteomic mapping of cytosol-facing outer mitochondrial and ER membranes in living human cells by proximity biotinylation. *Elife* **6**, (2017).
5. Friedman, J. R. *et al.* ER tubules mark sites of mitochondrial division. *Science (80-.).* **334**, 358–362 (2011).
6. Wong, Y. C., Ysselstein, D. & Krainc, D. Mitochondria-lysosome contacts regulate mitochondrial fission via RAB7 GTP hydrolysis. *Nature* **554**, 382–386 (2018).
7. Meneses-Salas, E. *et al.* Annexin A6 modulates TBC1D15/Rab7/StARD3 axis to control endosomal cholesterol export in NPC1 cells. *Cell. Mol. Life Sci.* **77**, 2839–2857 (2020).
8. Cipolat, S., De Brito, O. M., Dal Zilio, B. & Scorrano, L. OPA1 requires mitofusin 1 to promote mitochondrial fusion. *Proc. Natl. Acad. Sci. U. S. A.* **101**, 15927–15932 (2004).
9. Song, Z., Ghochani, M., McCaffery, J. M., Frey, T. G. & Chan, D. C. Mitofusins and OPA1 mediate sequential steps in mitochondrial membrane fusion. *Mol. Biol. Cell* **20**, 3525–3532 (2009).
10. Hu, C. *et al.* OPA1 and MICOS Regulate mitochondrial crista dynamics and formation. *Cell Death Dis.* **11**, 1–17 (2020).
11. Lee, J. E., Westrate, L. M., Wu, H., Page, C. & Voeltz, G. K. Multiple dynamin family members collaborate to drive mitochondrial division. *Nature* **540**, 139–143 (2016).
12. Chakrabarti, R. *et al.* INF2-mediated actin polymerization at the ER stimulates mitochondrial calcium uptake, inner membrane constriction, and division. *J. Cell Biol.* **217**, 251–268 (2018).
13. Qin, J. *et al.* ER-mitochondria contacts promote mtDNA nucleoids active transportation via mitochondrial dynamic tubulation. *Nat. Commun.* **11**, (2020).
14. Modi, S. *et al.* Miro clusters regulate ER-mitochondria contact sites and link cristae organization to the mitochondrial transport machinery. *Nat. Commun.* **10**, 1–15 (2019).
15. Honrath, B. *et al.* Glucose-regulated protein 75 determines ER–mitochondrial coupling and sensitivity to oxidative stress in neuronal cells. *Cell Death Discov.* **3**, 1–13 (2017).
16. De vos, K. J. *et al.* VAPB interacts with the mitochondrial protein PTPIP51 to regulate calcium homeostasis. *Hum. Mol. Genet.* **21**, 1299–1311 (2012).
17. Stoica, R. *et al.* ER-mitochondria associations are regulated by the VAPB-PTPIP51 interaction and are disrupted by ALS/FTD-associated TDP-43. *Nat. Commun.* **5**, (2014).
18. Gomez-Suaga, P. *et al.* The ER-Mitochondria Tethering Complex VAPB-PTPIP51 Regulates Autophagy. *Curr. Biol.* **27**, 371–385 (2017).
19. Iwasawa, R., Mahul-Mellier, A. L., Datler, C., Pazarentzos, E. & Grimm, S. Fis1 and Bap31 bridge the mitochondria-ER interface to establish a platform for apoptosis induction. *EMBO J.* **30**, 556–568 (2011).
20. Simmen, T. *et al.* PACS-2 controls endoplasmic reticulum–mitochondria communication and Bid-mediated apoptosis. *EMBO J.* **24**, 717–729 (2005).

21. Namba, T. *et al.* CDIP1-BAP31 Complex Transduces Apoptotic Signals from Endoplasmic Reticulum to Mitochondria under Endoplasmic Reticulum Stress. *Cell Rep.* **5**, 331–339 (2013).

22. Namba, T. BAP31 regulates mitochondrial function via interaction with Tom40 within ER-mitochondria contact sites. *Sci. Adv.* **5**, 1–12 (2019).

23. Hamasaki, M. *et al.* Autophagosomes form at ER-mitochondria contact sites. *Nature* **495**, 389–393 (2013).

24. Moulis, M. *et al.* The Multifunctional Sorting Protein PACS-2 Controls Mitophagosome Formation in Human Vascular Smooth Muscle Cells through Mitochondria-ER Contact Sites. *Cells* **8**, 638 (2019).

25. Diao, J. *et al.* ATG14 promotes membrane tethering and fusion of autophagosomes to endolysosomes. *Nature* **520**, 563–566 (2015).

26. Tabara, L. C. & Escalante, R. VMP1 establishes ER-microdomains that regulate membrane contact sites and autophagy. *PLoS One* **11**, (2016).

27. Zhao, Y. G. *et al.* The ER-Localized Transmembrane Protein EPG-3/VMP1 Regulates SERCA Activity to Control ER-Isolation Membrane Contacts for Autophagosome Formation. *Mol. Cell* **67**, 974-989.e6 (2017).

28. Wang, P., Kou, D. & Le, W. Roles of VMP1 in Autophagy and ER–Membrane Contact: Potential Implications in Neurodegenerative Disorders. *Front. Mol. Neurosci.* **13**, (2020).

29. Murphy, S. E. & Levine, T. P. VAP, a Versatile Access Point for the Endoplasmic Reticulum: Review and analysis of FFAT-like motifs in the VAPome. *Biochim. Biophys. Acta - Mol. Cell Biol. Lipids* **1861**, 952–961 (2016).

30. Eisenberg-Bord, M., Shai, N., Schuldiner, M. & Bohnert, M. A Tether Is a Tether Is a Tether: Tethering at Membrane Contact Sites. *Dev. Cell* **39**, 395–409 (2016).

31. Prinz, W. A., Toulmay, A. & Balla, T. The functional universe of membrane contact sites. *Nature Reviews Molecular Cell Biology* vol. 21 7–24 (2020).

32. Scorrano, L. *et al.* Coming together to define membrane contact sites. *Nat. Commun.* **10**, 1–11 (2019).

33. Di Mattia, T. *et al.* FFAT motif phosphorylation controls formation and lipid transfer function of inter-organelle contacts. *EMBO J.* **39**, e104369 (2020).

34. Di Mattia, T. *et al.* Identification of MOSPD2, a novel scaffold for endoplasmic reticulum membrane contact sites. *EMBO Rep.* **19**, 1–22 (2018).

35. Alpy, F. *et al.* STARD3 or STARD3NL and VAP form a novel molecular tether between late endosomes and the ER. *J. Cell Sci.* **126**, 5500–5512 (2013).

36. Wilhelm, L. P. *et al.* STARD 3 mediates endoplasmic reticulum-to-endosome cholesterol transport at membrane contact sites . *EMBO J.* **36**, 1412–1433 (2017).

37. Höglinger, D. *et al.* NPC1 regulates ER contacts with endocytic organelles to mediate cholesterol egress. *Nat. Commun.* **10**, 1–14 (2019).

38. Eden, E. R. *et al.* Annexin A1 Tethers Membrane Contact Sites that Mediate ER to Endosome Cholesterol Transport. *Dev. Cell* **37**, 473–483 (2016).

39. Rocha, N. *et al.* Cholesterol sensor ORP1L contacts the ER protein VAP to control Rab7-RILP-p150Glued and late endosome positioning. *J. Cell Biol.* **185**, 1209–1225 (2009).

40. Wijdeven, R. H. *et al.* Cholesterol and ORP1L-mediated ER contact sites control autophagosome transport and fusion with the endocytic pathway. **7**, 1–14 (2016).

41. Du, X. *et al.* A role for oxysterol-binding protein-related protein 5 in endosomal cholesterol trafficking. *J. Cell Biol.* **192**, 121–135 (2011).

42. Chung, J. *et al.* PI4P/phosphatidylserine countertransport at ORP5- and ORP8-mediated ER - Plasma membrane contacts. *Science (80-.).* **349**, 428–432 (2015).

43. Raiborg, C. *et al.* Repeated ER-endosome contacts promote endosome translocation and neurite outgrowth. *Nature* **520**, 234–238 (2015).

44. Elbaz-Alon, Y. *et al.* PDZD8 interacts with Protrudin and Rab7 at ER-late endosome membrane contact sites associated with mitochondria. *Nat. Commun.* **11**, 1–14 (2020).

45. Guillén-Samander, A., Bian, X. & de Camilli, P. PDZD8 mediates a Rab7-dependent interaction of the ER with late endosomes and lysosomes. *Proc. Natl. Acad. Sci. U. S. A.* **116**, 22619–22623 (2019).

46. Bagchi, P., Torres, M., Qi, L. & Tsai, B. Selective EMC subunits act as molecular tethers of intracellular organelles exploited during viral entry. *Nat. Commun.* **11**, 1–15 (2020).

47. Hoyer, M. J. *et al.* A Novel Class of ER Membrane Proteins Regulates ER-Associated Endosome Fission. *Cell* **175**, 254-265.e14 (2018).

48. Wu, H. & Voeltz, G. K. Reticulon-3 Promotes Endosome Maturation at ER Membrane Contact Sites. *Dev. Cell* **56**, 52-66.e7 (2021).

49. Eden, E. R., White, I. J., Tsapara, A. & Futter, C. E. Membrane contacts between endosomes and ER provide sites for PTP1B-epidermal growth factor receptor interaction. *Nat. Cell Biol.* **12**, 267–272 (2010).

50. Peretti, D., Dahan, N., Shimoni, E., Hirschberg, K. & Lev, S. Coordinated lipid transfer between the endoplasmic reticulum and the golgi complex requires the VAP proteins and is essential for Golgi-mediated transport. *Mol. Biol. Cell* **19**, 3871–3884 (2008).

51. Jing, J. *et al.* Proteomic mapping of ER-PM junctions identifies STIMATE as a regulator of Ca 2+ influx. *Nat. Cell Biol.* **17**, 1339–1347 (2015).

52. Liou, J., Fivaz, M., Inoue, T. & Meyer, T. Live-cell imaging reveals sequential oligomerization and local plasma membrane targeting of stromal interaction molecule 1 after Ca2+ store depletion. *Proc. Natl. Acad. Sci. U. S. A.* **104**, 9301–9306 (2007).

53. Giordano, F. *et al.* PI(4,5)P2-Dependent and Ca2+-Regulated ER-PM interactions mediated by the extended synaptotagmins. *Cell* **153**, 1494 (2013).

54. Saheki, Y. *et al.* Control of plasma membrane lipid homeostasis by the extended synaptotagmins. *Nat. Cell Biol.* **18**, 504–515 (2016).

55. Chang, C. L. & Liou, J. Phosphatidylinositol 4, 5-bisphosphate homeostasis regulated by Nir2 and Nir3 proteins at endoplasmic reticulum-plasma membrane junctions. *J. Biol. Chem.* **290**, 14289–14301 (2015).

56. Hua, R. *et al.* VAPs and ACBD5 tether peroxisomes to the ER for peroxisome maintenance and lipid homeostasis. *J. Cell Biol.* **216**, 367–377 (2017).

57. Costello, J. L. *et al.* ACBD5 and VAPB mediate membrane associations between peroxisomes and the ER. *J. Cell Biol.* **216**, 331–342 (2017).

58. Costello, J. L., Castro, I. G., Schrader, T. A., Islinger, M. & Schrader, M. Peroxisomal ACBD4 interacts with VAPB and promotes ER-peroxisome associations. *Cell Cycle* **16**, 1039–1045 (2017).

59. Chang, C. L. *et al.* Spastin tethers lipid droplets to peroxisomes and directs fatty acid trafficking through ESCRT-III. *J. Cell Biol.* **218**, 2583–2599 (2019).

60. Harner, M. *et al.* The mitochondrial contact site complex, a determinant of mitochondrial architecture. *EMBO J.* **30**, 4356–4370 (2011).

61. Li, H. *et al.* Mic60/Mitofilin determines MICOS assembly essential for mitochondrial dynamics and mtDNA nucleoid organization. *Cell Death Differ.* **23**, 380–392 (2016).

62. Tirrell, P. S., Nguyen, K. N., Luby-Phelps, K. & Friedman, J. R. MICOS subcomplexes assemble

independently on the mitochondrial inner membrane in proximity to ER contact sites. *J. Cell Biol.* **219**, (2020).

63. Weber, T. A. *et al.* APOOL Is a Cardiolipin-Binding Constituent of the Mitofilin/MINOS Protein Complex Determining Cristae Morphology in Mammalian Mitochondria. *PLoS One* **8**, e63683 (2013).

64. Xie, J., Marusich, M. F., Souda, P., Whitelegge, J. & Capaldi, R. A. The mitochondrial inner membrane protein Mitofilin exists as a complex with SAM50, metaxins 1 and 2, coiled-coil-helix coiled-coil-helix domain-containing protein 3 and 6 and DnaJC11. *FEBS Lett.* **581**, 3545–3549 (2007).

65. Ding, C. *et al.* Mitofilin and CHCHD6 physically interact with Sam50 to sustain cristae structure. *Sci. Rep.* **5**, 16064 (2015).

66. Hoppins, S. *et al.* A mitochondrial-focused genetic interaction map reveals a scaffold-like complex required for inner membrane organization in mitochondria. *J. Cell Biol.* **195**, 323–340 (2011).

67. An, J. *et al.* CHCM1/CHCHD6, novel mitochondrial protein linked to regulation of mitofilin and mitochondrial cristae morphology. *J. Biol. Chem.* **287**, 7411–7426 (2012).

68. PJ, T. *et al.* A subcellular map of the human proteome. *Science (80-.).* **356**, (2017).

69. Consortium, T. G. O. *et al.* Gene Ontology: tool for the unification of biology. *Nat. Genet.* **25**, 25 (2000).

70. Consortium, G. O. The Gene Ontology resource: enriching a GOld mine. *Nucleic Acids Res.* **49**, D325–D334 (2021).

71. Betsinger, C. N. *et al.* The human cytomegalovirus protein pUL13 targets mitochondrial cristae architecture to increase cellular respiration during infection. *Proc. Natl. Acad. Sci. U. S. A.* **118**, 1–12 (2021).

72. Haag, S. M. *et al.* Targeting STING with covalent small-molecule inhibitors. *Nature* **559**, 269–273 (2018).

73. Thomson, D. W. *et al.* Discovery of GSK8612, a Highly Selective and Potent TBK1 Inhibitor. *ACS Med. Chem. Lett.* **10**, 780–785 (2019).
