# Supplementary material for: Restructured membrane contacts rewire organelles for human cytomegalovirus infection
Source: Nat Commun. 2022 Aug 11;13:4720. doi: 10.1038/s41467-022-32488-6 (PMC9366835; doi:10.1038/s41467-022-32488-6)
Supplement: Supplementary file 3 — Description of Additional Supplementary Files [file 41467_2022_32488_MOESM3_ESM.pdf]

Description of Additional Supplementary Files

SUPPLEMENTARY MOVIES

Supplementary Movie 1. *3D reconstructions of ER-mitochondria interactions in human fibroblasts infected with HCMV.*

Confocal images of fixed HCMV-infected cells are labeled for ER (eGFP-Sec61β, cyan), mitochondria (mito-BFP, red), and the viral protein IE1 (antibody, magenta) and imaged as z-stacks with 0.2µm steps throughout the cell depth. Each zoom (7x7µm) is from the previous whole-cell stack. Scale is indicated in 10µm intervals at *right*.

Supplementary Movie 2. *Live-cell microscopy of ER-mitochondria dynamics across HCMV infection time.*

Live super-resolution movies of cells labeled for ER (eGFP-Sec61β, cyan) and mitochondria (mito-BFP, red), infected with HCMV, and imaged every 24 hours. After the 120hpi timepoint is also shown a movie of a 7x7µm zoom region from a cell labeled for ER (mCherry-Sec61β, cyan), mitochondria (mito-BFP, red), and the OMM (eGFP-OMP25, yellow), showing stable and asymmetric encapsulations. Movies are 2 minutes with 2 second intervals. Scale bars are 10µm.

Supplementary Movie 3. *ER and peroxisome interactions in live cells across the HCMV replication cycle.*

Live imaging of cells labeled for ER (eGFP-Sec61β, cyan) and peroxisomes (mCh-PTS1, red), 2 minutes with 2 second intervals at 0, 24, 48, 72, 96, and 120 hpi with HCMV. Scale bars are 10µm.

Supplementary Movie 4. *3D reconstruction of ER and peroxisomes in an HCMV-infected fibroblast cell at 120 hpi.*

Shown are fixed fibroblast cells in uninfected and 120 hpi states, labeled for ER (eGFP-Sec61β, cyan), peroxisome membranes (PEX14 antibody, red), and the viral protein IE1 (antibody, magenta) and imaged with 0.2µm steps throughout the cell depth. Each zoom (7x7µm) is from the previous whole-cell stack. Scale is indicated in 10µm intervals along right.

Supplementary Movie 5. *3D reconstructions of peroxisomes in control, ACBD5 KD, and ACBD5 OE cells before and 120 hours after HCMV infection.*

Fibroblast cells from control, ACBD5 KD, and ACBD5 OE genetic backgrounds stained for peroxisome membranes (PEX14 antibody, white) are displayed as reconstructed z-stacks, imaged with 0.2µm steps throughout the cell depth. Scale is indicated in 10µm intervals along right.

SUPPLEMENTARY DATASETS

Supplementary Data 1. *Library of peptides monitored for MCS-PRM analysis of organelle contact proteins, Related to Figure 1.*

Listed are MCS proteins, viral proteins as markers for infection, and internal loading control proteins included in our MCS-PRM assay, with the unique peptides per protein used for detection and quantification and their corresponding *m/z* precursor masses and charge values. See Excel file Supplementary Data 1.

Supplementary Data 2. *MCS-PRM datasets for infections with human viruses, Related to Figure 1.*

Each table (tabs within a single Excel file) shows the average peptide abundance for MCS proteins (scaled to the mean across timepoints). Also included are the standard deviations, CV values, and three-way ANOVA significance scores calculated across all peptides for all biological replicates. All raw data can be accessed via the PRIDE ProteomeXchange and Panorama repositories (see Data Availability section in manuscript for details). See Excel file Supplementary Data 2.

A. HCMV infectious cycle (0, 8, 24, 48, 72, 96, 120 hpi, N=6 biological replicates).

B. HSV-1 infectious cycle (0, 2, 6, 12, 18, 24 hpi, N=4 biological replicates).

C. Influenza A infectious cycle (0, 2, 6, 12, 18, 24 hpi, N=3 biological replicates).

D. HCoV-OC43 infectious cycle (0, 3, 6, 12 hpi, N=3 biological replicates).

Supplementary Data 3. *Comparison of MCS protein abundances to whole-proteome data for organelle localization and functional classifications.*

A. Listed are the high-confidence organelle marker proteins derived from the Human Protein Atlas[68] (http://www.proteinatlas.org/) and the proteins corresponding to Gene Ontology[69,70] classes relevant to MCS functions, used for the proteomic comparisons in Supplementary Figures 3-4. Corresponding protein abundance values for these marker proteins, and for each infection, are given in the following Supplementary Data 3B-D.

B. MCS-PRM data during HCMV infection compared to whole-proteome abundance changes, sorted by localization or function and using abundance values from the DDA-MS analysis by Jean Beltran *et al.*, *Cell Systems* 2016. Localization comparison is at *left*, and functional comparison is at *right*.

C. MCS-PRM data during HSV-1 infection compared to whole-proteome abundance changes, sorted by localization or function and using abundance values from the DDA-MS analysis by Lum *et al.*, *Cell Systems* 2018. Localization comparison is at *left*, and functional comparison is at *right*.

D. MCS-PRM data during Influenza A infection compared to whole-proteome abundance changes, sorted by localization or function and using abundance values from a DDA-MS analysis done in parallel to the MCS-PRM analyses in this study. Localization comparison is at *left*, and functional comparison is at *right*.

E. MCS-PRM data during HCoV-OC43 infection compared to whole-proteome abundance changes, sorted by localization or function and using abundance values from a DDA-MS analysis done in parallel to the MCS-PRM analyses in this study. Localization comparison is at *left*, and functional comparison is at *right*.
