# Supplementary material for: Restructured membrane contacts rewire organelles for human cytomegalovirus infection
Source: Nat Commun. 2022 Aug 11;13:4720. doi: 10.1038/s41467-022-32488-6 (PMC9366835; doi:10.1038/s41467-022-32488-6)
Supplement: Supplementary file 12 — Reporting Summary [file 41467_2022_32488_MOESM12_ESM.pdf]

nature portfolio

Corresponding author(s): Ileana M. Cristea

Last updated by author(s): Jun 16, 2022

Reporting Summary

Nature Portfolio wishes to improve the reproducibility of the work that we publish. This form provides structure for consistency and transparency in reporting. For further information on Nature Portfolio policies, see our Editorial Policies and the Editorial Policy Checklist.

Statistics

For all statistical analyses, confirm that the following items are present in the figure legend, table legend, main text, or Methods section.

n/a	Confirmed	
☐	☒	The exact sample size (*n*) for each experimental group/condition, given as a discrete number and unit of measurement
☐	☒	A statement on whether measurements were taken from distinct samples or whether the same sample was measured repeatedly
☐	☒	The statistical test(s) used AND whether they are one- or two-sided<br>*Only common tests should be described solely by name; describe more complex techniques in the Methods section.*
☐	☒	A description of all covariates tested
☐	☒	A description of any assumptions or corrections, such as tests of normality and adjustment for multiple comparisons
☐	☒	A full description of the statistical parameters including central tendency (e.g. means) or other basic estimates (e.g. regression coefficient) AND variation (e.g. standard deviation) or associated estimates of uncertainty (e.g. confidence intervals)
☐	☒	For null hypothesis testing, the test statistic (e.g. *F*, *t*, *r*) with confidence intervals, effect sizes, degrees of freedom and *P* value noted<br>*Give P values as exact values whenever suitable.*
☒	☐	For Bayesian analysis, information on the choice of priors and Markov chain Monte Carlo settings
☒	☐	For hierarchical and complex designs, identification of the appropriate level for tests and full reporting of outcomes
☒	☐	Estimates of effect sizes (e.g. Cohen's *d*, Pearson's *r*), indicating how they were calculated

Our web collection on statistics for biologists contains articles on many of the points above.

Software and code

Policy information about availability of computer code

Data collection	For proteomics acquisition: Xcalibur (ThermoFisher Scientific OPTON-30965); for microscopy acquisition: NIS Elements Advanced Research (Nikon); for western blot acquisition: Odyssey X (Li-Cor)
Data analysis	For proteomics analysis: Proteome Discoverer 2.4 (ThermoFisher Scientific OPTON-30957), Skyline (MacCoss Lab, University of Washington); for microscopy analysis: NIS Elements Advanced Research (Nikon), ImageJ (NIH); for western blotting: ImageJ (NIH)

For manuscripts utilizing custom algorithms or software that are central to the research but not yet described in published literature, software must be made available to editors and reviewers. We strongly encourage code deposition in a community repository (e.g. GitHub). See the Nature Portfolio guidelines for submitting code & software for further information.

Data

Policy information about availability of data

All manuscripts must include a data availability statement. This statement should provide the following information, where applicable:

- Accession codes, unique identifiers, or web links for publicly available datasets
- A description of any restrictions on data availability
- For clinical datasets or third party data, please ensure that the statement adheres to our policy

All MCS-PRM MS data generated in this study has been deposited to Panorama Public and can be accessed at the following URL: https://panoramaweb.org/mcsPRMviruses.url. The RAW data and a compiled MCS peptide library can also be found at PRIDE ProteomeXchange with the dataset identifier PXD023761.

Human research participants

Policy information about studies involving human research participants and Sex and Gender in Research.

Reporting on sex and gender	N/A
Population characteristics	N/A
Recruitment	N/A
Ethics oversight	N/A

Note that full information on the approval of the study protocol must also be provided in the manuscript.

Field-specific reporting

Please select the one below that is the best fit for your research. If you are not sure, read the appropriate sections before making your selection.

☒ Life sciences ☐ Behavioural & social sciences ☐ Ecological, evolutionary & environmental sciences

For a reference copy of the document with all sections, see nature.com/documents/nr-reporting-summary-flat.pdf

Life sciences study design

All studies must disclose on these points even when the disclosure is negative.

Sample size	For the targeted mass spectrometry analyses, 2-5 unique peptides were selected per proteins, according to guidelines for high quality protein quantification. All experiments were performed in a minimum of two biological replicates, with most quantitative assays performed in 3-6 biological replicates to ensure adequate statistical measurements. Sample sizes for all microscopy experiments were selected based on guidelines for phenotypic analyses as determined by peer-reviewed studies in the field, e.g., minimum of 10 mitochondria analyzed per cell in minimum 15 cells per sample/condition/timepoint/biological replicate.
Data exclusions	No data was excluded.
Replication	All experiments were performed in biological replicates of two or more, often using multiple approaches to confirm findings. The findings from quantitative mass spectrometry measurements were further confirmed using high-resolution and live-cell microscopy analyses, as well as western blotting. Similar results were obtained in every experiment, and all graphical representations (and supplementary datasets) of proteomics data in this manuscript is the combined report of all biological replicates. For microscopy analyses, experiments were performed independently at minimum three and up-to >20 times (e.g., for ER-mitochondria interactions with various additional parameters, such as PTPIP51 staining), with similar results obtained in every experiment. For every experiment included in our study, at least one biological replicate was obtained and analyzed from different cell batches at different times (e.g., months or years apart) to broaden the biological replication.
Randomization	For every genetic and infection tested, control (e.g., nontargeted siRNA or plasmid) or mock (i.e., uninfected) samples were analyzed in parallel to genetic perturbations (e.g., siRNA KD or plasmid OE) and infection timepoints (e.g., 24-120 hpi). Samples (e.g., tissue culture cells) pertaining to each group were randomly allocated prior to genetic or infection conditions, then clearly labeled and treated with the exact same parameters as all other samples (e.g., cell growth conditions, incubation times, lysis protocols, proteomic and microscopy acquisition and analysis guidelines) for the duration of the experiment. This is described in detail for each experiment in the Methods section.
Blinding	For infections and genetic perturbations, investigators were not blinded during sample collection, as these samples had to be differentially prepared (e.g., siRNA transfection or collection at a specific timepoint of infection) and clearly identified for future analyses. However, all experiments were performed and repeated by multiple scientists in the lab, for different biological replicates across years of experiments for this study. Wherever possible, the first and second authors rotated collecting versus analyzing proteomics and microscopy samples to limit bias from a single investigator.

Reporting for specific materials, systems and methods

We require information from authors about some types of materials, experimental systems and methods used in many studies. Here, indicate whether each material, system or method listed is relevant to your study. If you are not sure if a list item applies to your research, read the appropriate section before selecting a response.

Materials & experimental systems

n/a	Involved in the study
☐	☒ Antibodies
☐	☒ Eukaryotic cell lines
☒	☐ Palaeontology and archaeology
☒	☐ Animals and other organisms
☒	☐ Clinical data
☒	☐ Dual use research of concern

Methods

n/a	Involved in the study
☒	☐ ChIP-seq
☒	☐ Flow cytometry
☒	☐ MRI-based neuroimaging

Antibodies

Antibodies used

Host protein antibodies used: PTPIP51 (also known as RMDN3, 1:1000 for IF and PLA, 1:250 for WB, Sigma HPA009975); VAP-B (1:200 for IF and PLA, ProteinTech 66191-1-Ig); STING (1:400 for IF, Abcam Ab198950); ACBD5 (1:500 for IF, 1:150 for WB, Sigma HPA012145); PEX14 (1:500 for IF, Abcam ab183885); IRF3 phospho-S386 (1:1000 for WB, Abcam ab76493); TBK1 phospho-S172 (1:1000 for WB, Cell Signaling 5483S); IRF3 (1:1000 for WB, Abcam ab68481); TBK1 (1:500 for WB, Cell Signaling 3504S); PARP (1:500 for WB, Cell Signaling 9524); α-tubulin (1:5000 for WB, Sigma Aldrich T6199); GAPDH (1:4000 for WB, Cell Signaling D16H11-5174S); STING (Alexa Fluor 488 conjugate, 1:400 for IF, Abcam ab198950).

Viral protein antibodies used: IE1 (HCMV, 1:40 for IF, 1:100 for WB, gift from Dr. Thomas Shenk, Princeton University); pUL26 (HCMV, 1:100 for WB); pUL99 (HCMV, 1:40 for IF, gift from Dr. Thomas Shenk, Princeton University); ICP4 (HSV-1, 1:100 for IF, SantaCruz sc-69809); NP (Infl. A, 1:60 for IF, gift from Dr. Thomas Shenk, Princeton University); N (OC43, 1:100 for IF, 1:2000 for WB, Millipore Sigma MAB9013). Viral antibodies from the group of Dr. Thomas Shenk are available upon request to Dr. Ileana M. Cristea.

Other antibodies/stains: Alexa Fluor Plus IgG (H+L) highly cross-adsorbed secondary antibodies (1:2000 for IF, 1:10,000 for WB, ThermoFisher Scientific: Goat anti-Mouse 488 A11001, Goat anti-Mouse 568 A11019, Goat anti-Mouse 647 A32728, Goat anti-Mouse 800 A32730, Goat anti-Rabbit 488 A32731, Goat anti-Rabbit 568 A11011, Goat anti-Rabbit 647 A32733, Goat anti-Rabbit 680 A27042, Goat anti-Rabbit 800 A32735); DAPI (1:1000, ThermoFisher Scientific #62248).

Validation

All antibodies used are validated by the companies described above; specific papers and data are provided on the companies' websites (e.g., western blotting, immunofluorescence performed in KO backgrounds). We further validated the anti-PTPIP51 and anti-ACBD5 antibodies in knockdown backgrounds, using immunofluorescence and Western blotting, prior to proximity ligation analyses. The antibodies against viral proteins (from the group of Thomas Shenk) have been previously validated by immunoaffinity purification of the targeted proteins and analysis by mass spectrometry; in this study, we also validated these antibodies by western blotting, comparing infected to uninfected samples.

Eukaryotic cell lines

Policy information about cell lines and Sex and Gender in Research

Cell line source(s)

All experiments were performed in MRC5 human fibroblast cells (ATCC, CCL-171), except for experiments specific to HCoV-OC43, which were performed in RPTE epithelial cells (Lonza, CC-2553) and LLC-MK2 cells (ATTC, CCL-7).

Authentication

Each cell line used was certified by the company from which they were purchased, using short terminal repeat (STR) profiling. A certificate of analysis was included with each cell line.

Mycoplasma contamination

Each cell line used was confirmed to be mycoplasma free prior to shipment; mycoplasma testing is performed regularly in lab, confirming that all cells used were mycoplasma free for all experiments.

Commonly misidentified lines
(See ICLAC register)

No commonly misidentified lines were used in this study.
